# A comparison in species distribution model performance of succulents using key species and subsets of environmental predictors

**DOI:** 10.1002/ece3.8981

**Published:** 2022-06-06

**Authors:** Catherine E. Buckland, Andrew J. A. C. Smith, David S. G. Thomas

**Affiliations:** ^1^ School of Geography and the Environment University of Oxford Oxford UK; ^2^ Department of Plant Sciences University of Oxford Oxford UK; ^3^ Geography, Archaeology and Environmental Studies University of the Witwatersrand Johannesburg South Africa

**Keywords:** Crassulacean acid metabolism (CAM), *Euphorbia tirucalli*, Hellmann–Eberle quotient, *Opuntia ficus‐indica*, species distribution model (SDM), succulents

## Abstract

Identifying the environmental drivers of the global distribution of succulent plants using the Crassulacean acid metabolism pathway of photosynthesis has previously been investigated through ensemble‐modeling of species delimiting the realized niche of the natural succulent biome. An alternative approach, which may provide further insight into the fundamental niche of succulent plants in the absence of dispersal limitation, is to model the distribution of selected species that are globally widespread and have become naturalized far beyond their native habitats. This could be of interest, for example, in defining areas that may be suitable for cultivation of alternative crops resilient to future climate change. We therefore explored the performance of climate‐only species distribution models (SDMs) in predicting the drivers and distribution of two widespread CAM plants, *Opuntia ficus*‐*indica* and *Euphorbia tirucalli*. Using two different algorithms and five predictor sets, we created distribution models for these exemplar species and produced an updated map of global inter‐annual rainfall predictability. No single predictor set produced markedly more accurate models, with the basic bioclim‐only predictor set marginally out‐performing combinations with additional predictors. Minimum temperature of the coldest month was the single most important variable in determining spatial distribution, but additional predictors such as precipitation and inter‐annual precipitation variability were also important in explaining the differences in spatial predictions between SDMs. When compared against previous projections, an *a posteriori* approach correctly does not predict distributions in areas of ecophysiological tolerance yet known absence (e.g., due to biotic competition). An updated map of inter‐annual rainfall predictability has successfully identified regions known to be depauperate in succulent plants. High model performance metrics suggest that the majority of potentially suitable regions for these species are predicted by these models with a limited number of climate predictors, and there is no benefit in expanding model complexity and increasing the potential for overfitting.

## Graphical abstract

Using two different algorithms and five predictor sets, we created distribution models for two example succulent species and produced an updated map of global inter‐annual rainfall predictability. High model performance metrics suggest that the majority of potentially suitable regions for these species are predicted by these models with a limited number of climate predictors, and there is no benefit in expanding model complexity and increasing the potential for overfitting.

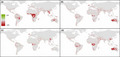

## 1 INTRODUCTION

Identifying the environmental conditions under which a species can thrive is an important question in biogeography and ecology both to understand the environmental tolerances of individual organisms and to be able to predict their distributions across current and future climates. Many parts of the world are likely to experience warmer climates and reduced and/or more variable precipitation in the decades ahead, so there is interest in determining which organisms may be relatively well adapted to these future climate regimes. A group of plants that are particularly characteristic of warm, semi‐arid parts of the world with strong seasonal rainfall patterns are succulents using the specific mode of photosynthesis known as Crassulacean acid metabolism (CAM). By virtue of being able to fix most of their carbon dioxide from the atmosphere at night rather than during the day time, CAM plants typically show high water‐use efficiency and can survive in environments with high daily temperatures and relatively limited water availability (Cushman, 2001; Lüttge, 2010; Osmond, 1978; Winter, 1985; Winter & Smith, 1996). The environmental resilience of these plants makes them attractive species for cultivation on marginal land for a variety of potential uses, such as fodder, bioethanol production, or as feedstock for anaerobic digestion (Acharya et al., 2019; Borland et al., 2009; Davis et al., 2011; Hastilestari et al., 2013; Holtum et al., 2011; Loke et al., 2011; Mason et al., 2015; Mwine et al., 2013; Yan et al., 2011). Such crops may be of particular value in semi‐arid regions most likely to experience increased drought risk (e.g., Marthews et al., 2019; Otto et al., 2018).

The growth and the ecophysiological controls on the natural distribution of CAM species have been widely studied and observed across a range of environments. Broadly speaking, the methods previously used to observe the distribution of specific CAM species can be split into those that are: observation based; growth/trial based; and those that are based on models—both process and data‐driven (Ringelberg et al., 2020). However, a comparison of the importance in different environmental parameters and derived indices in explaining the variability in CAM plant distribution has not yet been completed. Using existing studies published in the literature it is possible to compare areas of expected growth and productivity suitability (i.e., the locations with the environmental conditions required for specific species growth) (Guisan et al., 2017) based on process‐based models (e.g., Owen et al., 2015) or using climatic envelope methods (e.g., Louhaichi et al., 2015). However, there is also the potential to use methods based on derived environmental parameters and those driven by *a posteriori* models (e.g., species distribution modeling (Guisan et al., 2017)) to identify the relationship between known observations of CAM species and predictor variables; thus projecting maps of suitable biotic conditions for species to occur based on climatological, environmental, and/or biotic correlations (Aguirre‐Gutiérrez et al., 2013; Soberón & Nakamura, 2009).

Correlative species distribution models (SDMs) have been commonly employed as predictive tools to quantify relationships between species occurrence datasets and measurements of environmental variables (Dormann et al., 2012) across ecology, but seldom applied to the specific mapping of CAM plants. Equally, as noted by Bucklin et al. (2015), there remains no consensus on which variables should be included as predictors in SDM analysis more generally. While many climate‐only SDMs (i.e., using only climatic parameters) have been highlighted as important tools for both projecting current and future ecological niches (e.g., for guiding future conservation efforts (Elith & Leathwick, 2009) (Bucklin et al., 2015)), some studies have criticized this approach for providing only an incomplete representation of complex environmental systems (Araújo & Peterson, 2012; Bahn & McGill, 2007; Beale et al., 2008; Heikkinen et al., 2006). Using different combinations of bioclimatic and derived environmental indices, this study tests and compares the relative importance of parameters in explaining the distribution of CAM plants, focusing specifically on *Opuntia ficus*‐*indica* (L.) Mill. And *Euphorbia tirucalli* L. as example species. In doing so, this study attempts to define the best, minimal predictors of plant distribution so that models have the greatest predictive power without being over‐parameterized (Merow et al., 2014; Raes & Aguirre‐Gutiérrez, 2018).

Unlike recent analyses which have ensemble‐modeled numerous species with the aim of identifying the wider natural succulent biome distribution (e.g., Ringelberg et al., 2020), this study takes an alternative approach by selecting a minimal number of species of interest, but for which their distribution is successfully wide, occupying all available climatic niches, and with minimal dispersion limitations. There are numerous rare succulent species that have very restricted ranges on account of being dispersal‐limited for which this analysis would not be appropriate. By comparison, *O*. *ficus*‐*indica* is a successful invasive species having established itself across every continent (except Antarctica) (CABI, 2019) and found across all latitudes. *Opuntia ficus*‐*indica* and *E*. *tirucalli* have also shown great potential suitability for bioeconomic uses (Hastilestari et al., 2013; Mason et al., 2015); and are therefore suitable test species to use for this analysis which is interested in exploring the possibility for these plants to be actively grown as a crop—highlighting the potential that can be achieved with CAM plantation for bioeconomic and land restorative purposes. While most previous distribution modeling exercises have been built on the natural distribution of native species, additional novel information might be obtained from explicitly considering the extent and spread of introduced invasive species, once they are given the opportunity to spread into other parts of the “potential niche.”

Specifically, this study will compare different sets of variables to predict zones of potential suitability for *Opuntia ficus*‐*indica* and *Euphorbia tirucalli* growth. In doing so, this study aims to first predict the current locations with suitable biotic conditions for the occurrences of *O*. *ficus*‐*indica* and *E*. *tirucalli* using different SDMs tested in this study. Second, the results will help identify the most important set of variables that help define the environmental niche of two CAM species of interest. While the natural distribution of both species has generally been restricted to semi‐arid regions as outcompeted by other plants, their natural ecological requirements permit them growing in wetter areas, and competition factors have largely restricted the spread of the species to regions with annual rainfall <500 mm (Luttge, 2004). *Opuntia ficus*‐*indica* is a successful invasive which has been widely sighted across regions outside of central America (e.g., Africa, southern Europe), while *E*. *tirucalli* is native to Africa (Palgrave, 1977; Webb et al., 1984) but has also been found in central America, Europe, and other locations globally. Given the successful expansion, but different origins of these two species, comparison of the potential regions through which they could be successfully cultivated for bioeconomic (e.g., biogas) uses across a region (e.g., sub‐Saharan Africa) with low levels of energy access, increased agricultural pressure in the face of drought, and high climatic suitability for these species is particularly interesting (Buckland & Thomas, 2021). For this reason, this study will initially calibrate and project models based on a global view, before taking a deeper focus on Africa as a potential region for cultivation, bioenergy and bioeconomic uses.

## 2 MATERIALS AND METHODS

Using SDM techniques, this study compares the relative performance of five SDMs to predict the potential distribution of *O*. *ficus*‐*indica* and *E*. *tirucalli* based on current climatic conditions. The five SDMs each capture different combinations of environmental variables defined in the WorldClim 2.1 bioclim database (Fick & Hijmans, 2017) and derived indices or parameters that have previously been cited as impacting upon the spatial distribution of CAM plants: the Hellmann–Eberle quotient (a measure of inter‐annual rainfall predictability used by Ellenberg, 1981), the aridity index (the ratio between annual precipitation and potential evapotranspiration (PET)), cloud cover (as a proxy for light intensity), and the R‐index (the ratio between actual and PET) (Yao, 1974). As noted in Title and Bemmels (2018), the inclusion of more complex climatic indices may characterize environmental conditions that are more directly physiologically relevant to particular species than more primary climatic parameters (e.g., temperature, precipitation). Due to the successful invasive nature of both species, we have considered their expansion to be largely limited by environmental conditions rather than distribution‐limited, and thus only climatic‐based parameters have been used.

### 2.1 Predictor datasets

The choice of environmental variables selected should ideally be based on the known ecology of the species (Title & Bemmels, 2018), as this has previously demonstrated more realistic SDMs (Rödder et al., 2009; Saupe et al., 2012). With this in mind, a combination of bioclim datasets from the WorldClim 2.1 catalogue (Fick & Hijmans, 2017) and derived environmental metrics were compiled and a sensitivity analysis (Pearson’s Correlation) was used to remove highly correlated variables. Inclusion of co‐variant parameters leads to over‐parameterization of the model. All predictor datasets were bilinearly resampled to the same 2.5 min resolution.

#### 2.1.1 Bioclim datasets

Based on existing research of the parameters impacting the growth and distribution of succulents and CAM plants more generally (Acharya et al., 2019; Inglese & Scalenge, 2009; Le Houérou, 1996; Louhaichi et al., 2015; Masocha & Dube, 2018), and the results from covariance testing (Appendix A), four bioclim variables were selected for use as explanatory parameters (Table 1).

**TABLE 1:** Bioclim parameters (from Fick & Hijmans, 2017) used in the final model iterations

Bioclim variable	Environmental parameter
Bioclim 2	Mean diurnal temperature range (mean of monthly (max temp‐min temp)) (°C)
Bioclim 6	Minimum temperature of coldest month (°C)
Bioclim 12	Annual precipitation (mm)
Bioclim 15	Precipitation seasonality (coefficient of variation)

#### 2.1.2 Hellmann–Eberle quotient

The Hellmann–Eberle quotient provides a measure of inter‐annual precipitation variability and is defined as the ratio between precipitation of the wettest year and precipitation of the driest year over an extended period of time. Ellenberg (1981) examined the distribution pattern of tall stem succulents in relation to climate and found that they tended to occur in areas where rainfall was low (i.e., <500 mm per annum), but regularly received (i.e., where the Hellmann–Eberle quotient <5 over a series of years) (Cowling et al., 1997). Ellenberg’s original study from 1981 was based on 35 years of observations (1905‒1940) and has since been referred to and expanded in more recent studies exploring the controls on CAM distribution (e.g., Holtum et al., 2016, 2017; Lüttge, 2010; Ringelberg et al., 2020). Using historical monthly weather data from 1960 to 2018 AD from the CRU‐TS 4.03 dataset (Harris et al., 2014) downscaled with WorldClim 2.1 (Fick & Hijmans, 2017), we calculated a more recent version of the Hellmann–Eberle quotient based on annual historical precipitation levels at a 2.5 min spatial resolution (globally) to compare against observational occurrences of *O*. *ficus*‐*indica* and *E*. *tirucalli* from the Global Biodiversity Information Facility (GBIF.org, 2020). Individual GeoTiff files were analyzed and climate rasters were produced in R Studio (RStudio Team, 2019), before being combined with observational occurrence data in ArcGIS Pro 2.4.1.

Precipitation regime alone, however, is unlikely to explain the distribution of these species as it does not include the impact of minimum temperatures, which is known to be limiting for particular CAM species (Acharya et al., 2019; Herrando‐Moraira, 2020; Inglese & Scalenge, 2009; Smith et al., 2012; Stock et al., 1997). For this reason, combining the Hellmann–Eberle quotient with other bioclimatic parameters in the SDM analysis has the potential to improve our distributional understanding of key species of interest.

#### 2.1.3 Aridity index, R‐index and cloud cover

The Aridity Index (AI) is commonly considered to provide a measure of overall water availability, a central component to all vegetative growth. Based on global raster data from 1970 to 2000 AD, a global aridity index based upon the implementation of the Penman–Monteith reference evapotranspiration equation (Allen et al., 1998) was used in this study (Trabucco & Zomer, 2018). The R‐index is calculated as the ratio between actual evapotranspiration (AET) and PET and is a measure of plant water supply in relation to plant water demand (Yao, 1974). A global R‐index raster was calculated using the average annual AET and PET rates available via the Consultative Group for International Agricultural Research (Trabucco & Zomer, 2018). Finally, as a proxy for photosynthetically active radiation, cloud cover was included as a potential parameter that could be inversely related to plant growth. CAM plant growth shows a saturation‐type relationship to light intensity (Nobel, 1988; Nobel & Valenzuela, 1987) with the three main environmental limitations on CAM plant growth considered water, light, and temperature (Nobel, 1988; Owen et al., 2015). Process‐based models have thus included a proxy for light intensity as a measure to predict the variability in spatial productivity of CAM plant species in existing literature (e.g., Owen et al., 2015). In this study, a global raster of mean annual cloud cover based on 15 years (2000–2014 AD) of twice‐daily satellite observations was used from the EarthEnv data repository (Wilson & Jetz, 2016).

#### 2.1.4 Pearson’s correlation coefficient

A total of five combinations of environmental parameters and bioclim parameters (Fick & Hijmans, 2017) were used to model the relationship between environmental conditions and the observed distribution of *O*. *ficus*‐*indica* and *E*. *tirucalli* (Table 2). Prior to final environmental parameter selection for each of the five SDM combinations, Pearson’s correlation coefficient tests were conducted to test for covariance between the variables (Appendix A). Based on the results, and on an understanding of the main climatic parameters that influence CAM distribution, 4 bioclim variables were selected for use in the final model fitting (Table 1) alongside a combination of derived environmental indices.

### 2.2 Occurrence data

*Opuntia ficus*‐*indica* and *Euphorbia tirucalli* were the two species of interest selected for analysis in this study. The former is an especially suitable test species for this analysis since its occurrences are already occupying most of its geographic range allowing us to model a potential distribution closer to its fundamental niche (i.e., all the environmental conditions where a species could potentially exist) as opposed to the realized niche (i.e., those conditions in which the species currently does exist) (Chase & Leibold, 2003; Hutchinson, 1957). By comparison, often the current distributions of localized or very rare species are restricted by dispersal limitations and species interactions; in such cases the realized niche will be smaller than the fundamental niche, and we cannot independently test the impact of different climatic and environmental parameters on defining areas suitable for species occurrence.

**FIGURE 1:**
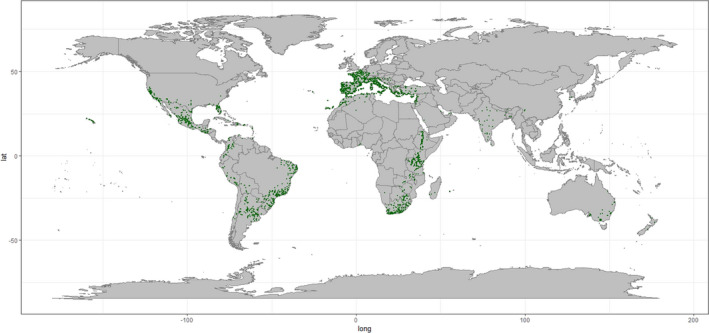
Final 2721 *Opuntia ficus*‐*indica* occurrences downloaded from the GBIF dataset (GBIF.org, 2020) after spatial bias analysis completed

**FIGURE 2:**
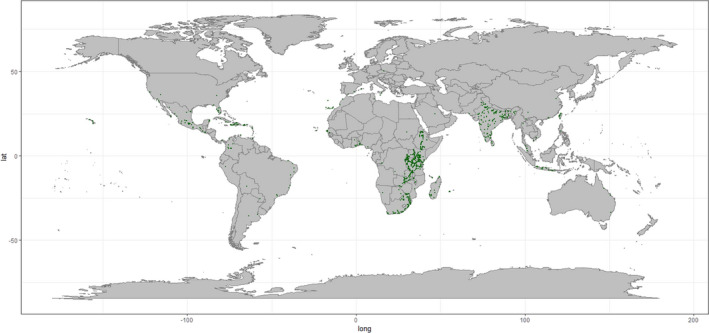
Final 1085 *Euphorbia tirucalli* occurrences downloaded from the GBIF dataset (GBIF.org, 2020) after spatial bias analysis completed

*Opuntia ficus*‐*indica* and *E*. *tirucalli* occurrence data were downloaded from the GBIF data repository (GBIF.org, 2020) (Accessed 09/06/2020) and cleaned according to the method described in Zizka (2019). Species occurrence data from both the native and introduced ranges was used for both species. One of the main aims of this study is to identify regions which could support the cultivation of these species under current climatic conditions (i.e., to map the fundamental niche of the species). As such, we do not need to limit the training dataset to the native distribution, rather observations of the species across a range of geographic zones are useful in identifying the scope of environmental settings which are suitable. Spatial bias of occurrence datasets has the potential to distort the interpretation of large‐scale biodiversity patterns (Ballesteros‐Mejia et al., 2013; Beck et al., 2014; Boakes et al., 2010; Varela et al., 2014; Yang et al., 2013), and SDMs are sensitive to the spatial bias of specimen records (Dudík & Phillips, 2005; Lintz et al., 2013; Phillips et al., 2009). Spatially biased data would have a two‐fold impact on distorting SDMs: first, through biasing the present data used to train and evaluate model performance (Hijmans et al., 2017); second in biasing the surface range envelope model used in the pseudo‐absence dataset generation (see below) and therefore model performance metrics. With this in mind, we applied a geographic sampling filter, selecting up to five occurrence data points from each 1° × 1° grid cell—reducing our datasets to 2721 and 1085 occurrences (from 8061 and 2313) of *O*. *ficus*‐*indica* and *E*. *tirucalli*, respectively (Figures 1 and 2).

### 2.3 Pseudo‐absences

Unlike “presence” datasets, “absence” datasets are not often readily available. Since some SDM algorithms require both datasets, pseudo‐absence (PA) datasets are created as a replacement for true absence records (Raes & Aguirre‐Gutiérrez, 2018). The use of PA data is widely accepted and has been shown in the SDM literature to be a useful approach to calibrate SDMs (Chefaoui & Lobo, 2008; Iturbide et al., 2018; Václavík & Meentemeyer, 2009; Wisz & Guisan, 2009). PA data are generated by sampling background areas from which presence records have not been identified through a range of different strategies, including: random, surface range envelope (SRE), or based on a minimum (or maximum) distance from known presence points. The sensitivity of SDM algorithms to the sample of PA when projecting under future climates varies between models and creates a source of SDM‐dependent uncertainty that should be considered when deciding on initial PA sampling and accounted for in SDM ensemble modeling (Iturbide et al., 2018).

Based on the recommendations of the findings in Barbet‐Massin et al. (2012) and Iturbide et al. (2018), an equal number of PAs were selected to presences with multiple PA realizations (five) to reduce overall uncertainty. Studies based on a single realization of PAs have the potential to mask results from poorly performing SDMs (Iturbide et al., 2018). Hence, five PA realizations were used to reduce the dependence on poorly performing SDMs and to ensure model fits were not dependent on a single realization where PAs have been biasedly generated from regions with few noted presences rather than few true presences (Barbet‐Massin et al., 2018). PAs were generated from all areas outside the suitable area estimated by a surface range envelope model (SRE) (Thuiller et al., 2014). SRE models are based on presence‐only data (Barbet‐Massin et al., 2012); SRE quantile refers to the quantile used to remove the most extreme values of each environmental variable for determining tolerance boundaries (quantile 0.025 ~ 95% confidence interval) (Hallgren et al., 2019).

### 2.4 Model fitting

There are numerous options for algorithms to use in SDM studies (summarized in Raes & Aguirre‐Gutiérrez, 2018), but there is often no model of “best” choice (Qiao et al., 2015). Fitting the data with the same algorithm over multiple repeats would yield different results, as would fitting the data across multiple algorithms. Overfitting occurs when an overly flexible model learns the noise in the training dataset to a level that negatively impacts the performance of the model when introduced to new input data. By comparison, inflexible models do not have the flexibility to fit complex relationships between parameters and predictor datasets. As such, inflexible models may not have the capacity to accurately fit the training dataset, nor to generalize well to new unseen data (e.g., projecting over a new time period or geographic location). In SDM, and machine learning more generally, we seek to find a balance in creating models with the capacity to fit variance but also avoid bias. Equally, defining “best” model is largely dependent on the choice of evaluative metric—for which there are numerous. Each evaluative metric measures a slightly different aspect of model performance, and thus while a model may perform well according to one measure, it may not be the “best” model according to another metric (Qiao et al., 2015; Raes & Aguirre‐Gutiérrez, 2018).

With this in mind, SDMs were initially fitted across two different algorithms which required the same PA dataset generation strategy: Boosted Regression Trees (Elith, 2008) (also known as Generalized Boosting Model GBM) and Random Forests (Breiman, 2001), before being combined in an ensemble model to obtain a consensus distribution (Marmion et al., 2009). Default model parameters found in the biomod2 package (Georges & Thuiller, 2013; Thuiller et al., 2014) were used and 10 repeats were completed per algorithm per PA selection, producing a total of 100 individual model repeats used for each ensemble model (a total of 500 individual model repeats across all five SDM scenarios). The between‐ and within‐modeling variability shown in SDM outputs has led to the widespread usage of ensemble models (Marmion et al., 2009; Qin et al., 2020; Raes & Aguirre‐Gutiérrez, 2018; Senay et al., 2013); capturing the uncertainty in model predictions across the different SDM algorithm outputs (Araújo & New, 2007; Dormann, 2018; Hao et al., 2019; Raes & Aguirre‐Gutiérrez, 2018), producing more consistent predictions when projecting new unseen data (e.g., future climate scenarios).

There are many strategies that can be used to combine predictions from individual models into an ensemble model. Following the recommendation of Hao et al. (2019), we have taken a more sophisticated approach which involved weighting the models based on their individual predictive performances. The performance of each individually trained model was assessed, and ensemble models were produced based on the true skill statistic (TSS) and relative operating characteristic (ROC) of each individual model (based on thresholds defined in Qin et al. (2020): those with ROC >0.5 imply that the model performed better than random). TSS metrics are widely used as a measure of relative performance in SDM studies and have been recommended over the use of other methods such as Kappa (Allouche et al., 2006). The TSS is calculated as: *Specificity* +*Sensitivity –1*, whereby “specificity” refers to the proportion of correctly predicted absences, and “sensitivity” refers to the proportion of correctly predicted presences. Individual models were combined using two ensemble‐model algorithms: weighted mean of probabilities and coefficient of variation of probabilities, to provide a measure of uncertainty in the former ensemble model. Current occurrence and predictor datasets were split 60% for training and validation, with the remaining 40% used for testing and evaluating model performance. All models were fitted and projected using the biomod2 package version 3.3 (Thuiller et al., 2014) in R Studio version 1.2.5033 (RStudio Team, 2019).

### 2.5 Evaluating model comparison

As well as TSS and AUC (ROC) scores calculated for each of the individual models, the TSS and AUC scores of the ensemble models were compared to determine the relative best performing model and identify whether the additional parameters used in SDMs 2–5 increased the predictive accuracy of SDM 1 (bioclim‐only predictors). As discussed in Komac et al. (2016), the AUC provides us with a measure of the performance of ordinal score models and a threshold measure of accuracy (Thuiller et al., 2005), while the TSS score provides us with a measure of evaluative performance which has all the advantages associated with the Cohen’s kappa statistic (Cohen, 1968) but is not sensitive to prevalence (Allouche et al., 2006). Ensemble models from the five SDM scenarios were initially projected on to the world to generate a continuous map showing variations in the suitability/probability of occurrence for the two species of interest. Then, using the ensemble model cut‐off values to provide a binary measure of habitat suitability, projections were then compared against projections based on existing methods from the literature (e.g., Louhaichi et al., 2015) to identify the spatial variability in identified suitable regions between the methods. Ensemble binary cut‐off values are calculated as those that give the maximum “sensitivity” and “specificity” scores (Thuiller et al., 2005).

### 2.6 Assessing variable importance

Individual variable importance was approximated using the Variables_importance function of the “biomod2” package (Thuiller et al., 2014). Variable importance was assessed for each of the five ensemble models and across each of the two species with the aim of determining which climatic or environmental factors have stronger effects on the species suitability across the region of interest. The principle of the biomod2 variable importance algorithm is to shuffle a single variable of the given data and produce model predictions with this new “shuffled” dataset. A Pearson’s correlation between the reference predictions and “shuffled” dataset predictions is calculated, with higher values corresponding to a greater influence the individual variable has on the model (i.e., a value of 0 assumes no influence of the variable on the model). Variable importance results were standardized across all predictors used per model and presented in percentage terms.

## 3 RESULTS

### 3.1 Ensemble model projections and comparisons

**FIGURE 3:**
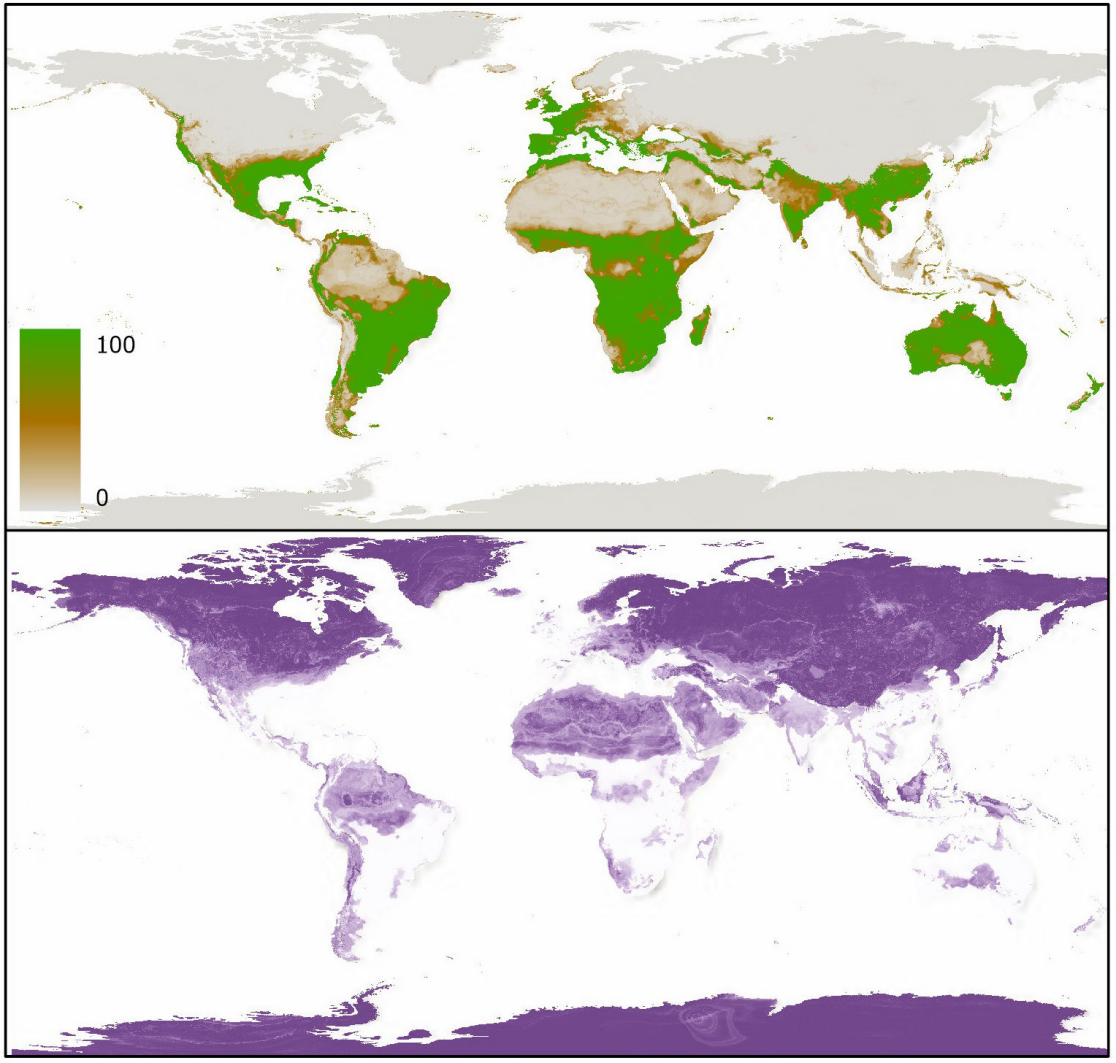
Species distribution model scenario 1 projection and uncertainty (coefficient of variation) based on occurrences of *O*. *ficus*‐*indica*

**FIGURE 4:**
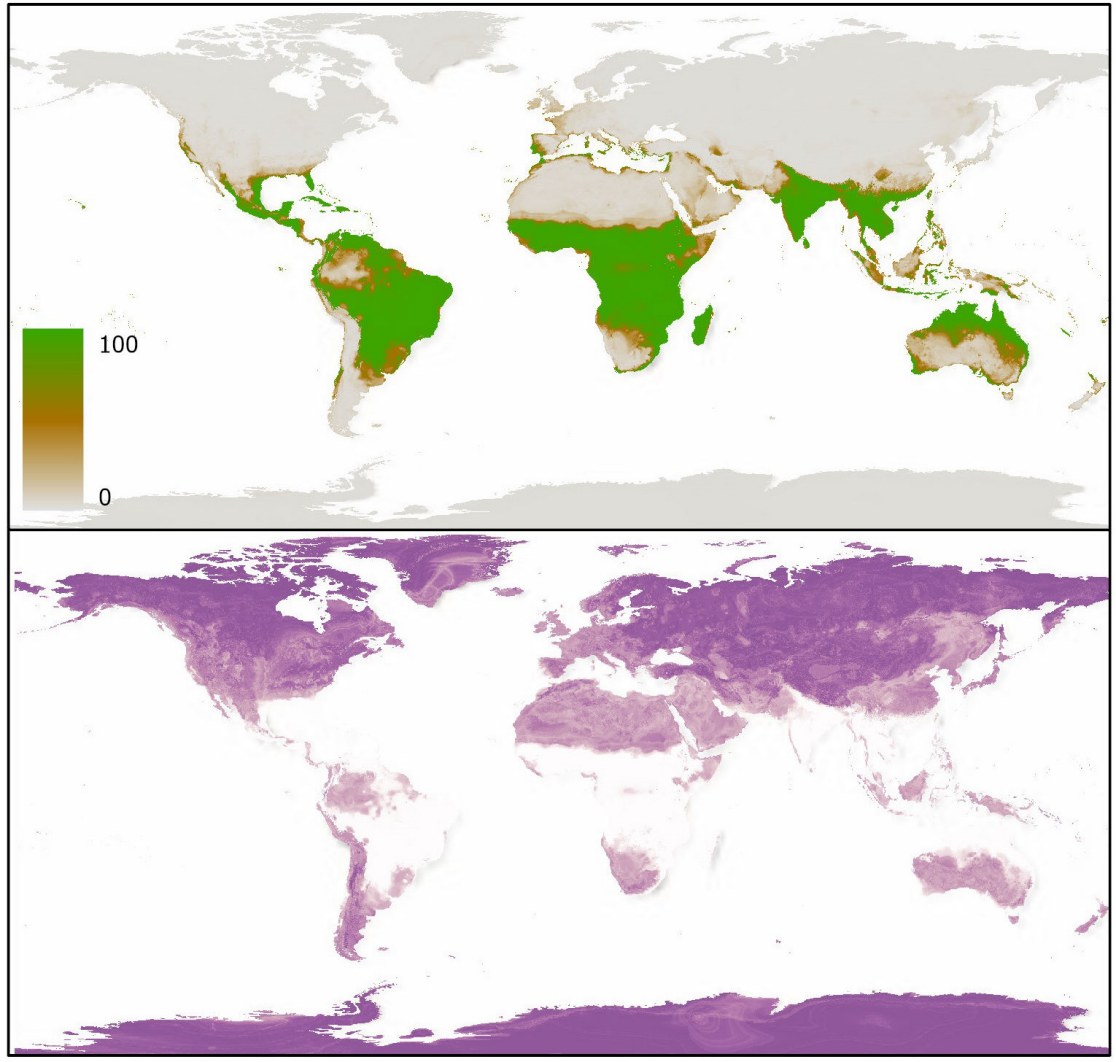
Species distribution model scenario 1 projection and uncertainty (coefficient of variation) based on occurrences of *E*. *tirucalli*

A total of 500 individual models and projections were produced for each species and ensembled to produce weighted mean projections with coefficient of variation (uncertainty between the individual projections) measurements for each of the scenarios. Ensemble results from SDM scenario 1 are presented in Figures 3 and 4, with TSS scores across the five ensembles shown in Table 3. SDM scenarios 2–5 are shown in Appendix E.

**TABLE 3:** Ensemble species distribution model (SDM) evaluative metrics (true skill statistic (TSS) and relative operating characteristic (ROC)) for each of the five *O*. *ficus*‐*indica* and *E*. *tirucalli* ensembled SDM scenarios. See Supplementary Information for binary cut‐off, Specificity, and Sensitivity scores

SDM scenario	*O. ficus indica*		*E. tirucalli*	
	TSS	ROC	TSS	ROC
1	0.930	0.997	0.955	0.998
2	0.914	0.994	0.932	0.996
3	0.916	0.995	0.948	0.997
4	0.925	0.996	0.954	0.998
5	0.918	0.995	0.949	0.997

Across both species, results show relatively little difference in the evaluative performance between the ensemble models when tested against the remaining 40% of the dataset, however, SDM 1 outperformed the other four SDMs for both *O*. *ficus*‐*indica* and *E*. *tirucalli* distribution projections (Table 3, Figures S7 and S8). The random forest algorithm generally performed best for *O*. *ficus*‐*indica* projections in both TSS and ROC scores, while GBMs marginally outperformed random forest models in the *E*. *tircualli* predictions (See Supplementary Information). Among both species and predictor scenarios, all models performed well with overall TSS scores >0.91 across all ensembles (Table 3). TSS scores for individual model performances showed high performance with little variability, ranging from 0.85 to 0.942 and from 0.87 to 0.968 for *O*. *ficus*‐*indica* and *E*. *tirucalli*, and 0.981–0.996 and 0.984–0.999 ROC scores, respectively (Tables S2 and S3). Due to the overall high performance of the individual models, all individual projections were included in the weighted ensemble model.

At a global scale, ensemble models across all five predictor scenarios indicated that both species have potential distributional ranges in the tropics and mid‐latitudes. The areas predicted most suitable for *O*. *ficus*‐*indica* include sub‐Saharan Africa, Mediterranean Europe, Australia, South America (especially Brazil and north‐eastern Argentina), central America and countries in southern and eastern Asia (e.g., India, China, and Thailand). Meanwhile the areas predicted most suitable for *E*. *tirucalli* growth are more restricted to the tropics, especially sub‐Saharan Africa, Brazil and northern South America, India, northern Australia and south China. The higher latitudes and hyper‐arid Sahara were predicted unsuitable for both species.

**FIGURE 5:**
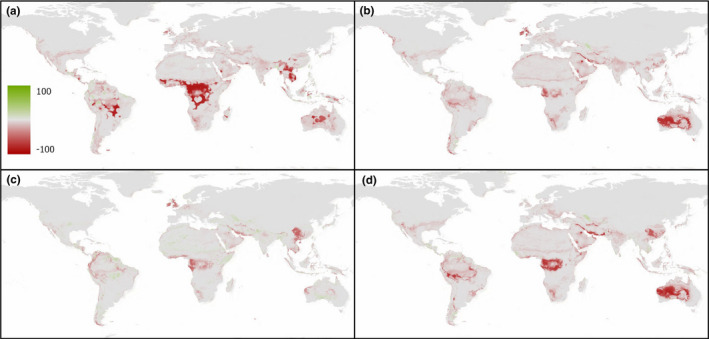
Deviation of species distribution model (SDM) scenarios 2–5 (a–d) from the results of the bioclim‐only scenario (SDM 1) for *O*. *ficus*‐*indica*. Red shading indicates areas where the relative SDM predicts a lower probability of *O*. *ficus*‐*indica* growth versus SDM 1, while green shading predicts areas with a higher probability of *O*. *ficus*‐*indica* projected occurrence

**FIGURE 6:**
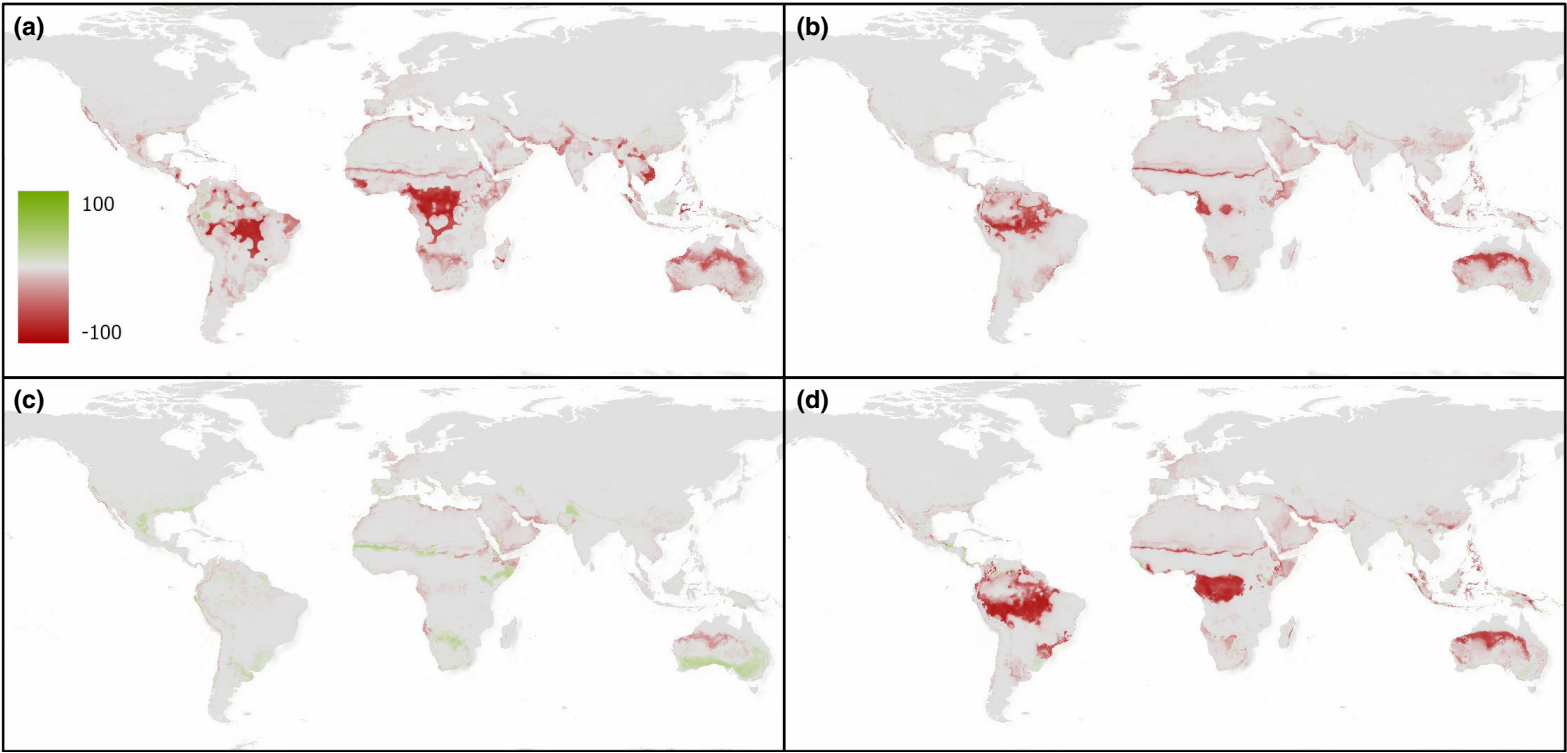
Deviation of species distribution model (SDM) scenarios 2–5 (a–d) from the results of the bioclim‐only scenario (SDM 1) for *E*. *tirucalli*. Red shading indicates areas where the relative SDM predicts a lower probability of *E*. *tirucalli* growth versus SDM 1, while green shading predicts areas with a higher probability of *E*. *tirucalli* projected occurrence

When the deviation in environmental suitability is compared between SDM scenarios (Figures 5 and 6), the inclusion of either the Hellmann–Eberle quotient, aridity index, or R‐index all produced overall results with lower suitability projections than those predicted using bioclim variables alone (SDM 1). It is only in SDM 4 (Figures 5c and 6c) that ensemble model projections suggest that some regions (typically those with reduced overall certainty) have a higher level of environmental suitability than projections based on the four bioclim variables alone. However, these results are not necessarily corroborated when we consider the binary cutoff values at a regional scale for example (i.e., where maximum specificity and sensitivity are achieved) and the results are presented as either “suitable” or “unsuitable” areas (Table 4). For example, results from the continuous profiles suggest SDM 4 estimates some areas of both increased and decreased suitability relative to SDM 1, yet the results from the binary cutoff values for the African continent suggest this projection produces the second lowest levels of regions suitable for *O*. *ficus*‐*indica* growth. By comparison, SDM 4 produces the largest suitable area estimates for the *E*. *tirucalli* projections, as well as demonstrating increased estimated suitability values in the continuous dataset for SDM 4 relative to SDM 1.

**TABLE 4:** Example total suitable area (million km^2^) calculations across the African continent (as an example) for *O*. *ficus*‐*indica* and *E*. *tirucalli* per species distribution model (SDM) scenario based on the binary cutoff values

SDM scenario	*O. ficus‐indica*	*E. tirucalli*
1	15.6	17.0
2	11.4	13.2
3	14.8	16.3
4	14.4	17.4
5	13.4	14.9

### 3.2 Environmental variable importance

Results from individual variable importance analysis were calculated based on the weighted mean ensemble models for each of the five SDM scenarios and across the two species of interest (Tables 5 and 6). Across both *O*. *ficus*‐*indica* and *E*. *tirucalli*, the minimum temperature of the coldest month shows a significantly higher variable importance factor than any of the other environmental parameters across the SDM scenarios. Equally, both species show similarity in response to annual precipitation, which demonstrates second greatest individual variable importance, except for when modeled in scenarios 3 and 5—when the Aridity index and R‐index, respectively, show high levels of variable importance and a reduction in the relative importance of annual precipitation.

**TABLE 5:** Standardized mean variable importance of each parameter across the five different species distribution model (SDM) scenarios for *O*. *ficus*‐*indica*

SDM scenario	Environmental predictors							
	Mean diurnal temp range	Min temp of coldest month	Annual precipitation	Precipitation seasonality	Hellmann–Eberle quotient	Aridity index	Cloud cover	R‐index
1	2%	74%	20%	4%	n/a	n/a	n/a	n/a
2	1%	70%	17%	4%	8%	n/a	n/a	n/a
3	2%	74%	5%	4%	n/a	15%	n/a	n/a
4	1%	78%	13%	5%	n/a	n/a	4%	n/a
5	1%	75%	5%	4%	n/a	n/a	n/a	14%

**TABLE 6:** Standardized mean variable importance of each parameter across the five different species distribution model (SDM) scenarios for *E*. *tirucalli*

SDM scenario	Environmental predictors							
	Mean diurnal temp range	Min temp of coldest month	Annual precipitation	Precipitation seasonality	Hellmann Eberle quotient	Aridity index	Cloud cover	R‐index
1	2%	71%	26%	1%	n/a	n/a	n/a	n/a
2	2%	68%	23%	1%	7%	n/a	n/a	n/a
3	2%	74%	3%	1%	n/a	19%	n/a	n/a
4	1%	82%	14%	1%	n/a	n/a	2%	n/a
5	2%	76%	4%	1%	n/a	n/a	n/a	18%

## 4 DISCUSSION

### 4.1 Drivers of CAM plant distribution

Results from the ensemble model evaluative performance and individual variable importance analysis suggest that for both species there is not any overall model improvement with the inclusion of either the aridity index, Hellmann–Eberle quotient, cloud cover conditions or R‐index (i.e., SDMs 2–5) over the primary four bioclim variables (SDM1); and that the dominant variable of importance in explaining the spatial variability in ecological niche is the minimum temperature of the coldest month. With this in mind, it seems there is little benefit in the inclusion of additional predictors beyond the four bioclim parameters, regardless of which additional parameters were to be considered. With results not differing significantly between the SDM scenario analyses, it suggests that the most important bioclimate predictors (SDM 1) primarily shaped the patterns across all models produced. These results of variable importance are in agreement with von Willert et al. (1992) who consider low temperatures a key limiting factor in succulent growth when referring to succulent growth on hill slopes in Tenerife. The relatively minor variation in overall model performance between the SDMs with and without the additional parameters is also in agreement with the results noted by Bucklin et al. (2015), who have suggested that climate‐only predictor sets may be equally as effective in producing environmental suitability maps.

Following the role of extreme cold temperatures, moisture availability measured either through annual precipitation or the aridity index or R‐index is shown to be the second most important independent variable on overall model performance. When an alternative precipitation metric is included in the model (i.e., SDM scenarios 3 and 5), the relative importance of annual precipitation is reduced. The compound variable, aridity index, is defined as the ratio between annual precipitation and PET—reflecting the amount of moisture potentially available for vegetation growth. Equally, the R‐index as calculated as the ratio between AET and PET, provides a measure of water supply in relation to water demand (Yao, 1974); unsurprising that the relative importance of annual precipitation as an individual metric is reduced when considered alongside these compound variables. However, it is also worth noting that the R‐index used in this study (derived from AET and PET datasets (Trabucco & Zomer, 2018)) is based on spatially standardized vegetation and soil coefficients (i.e., based on typical agronomic crops at maturity and an average soil texture for plant rooting depth at 2 m). Variations in both the vegetation and soil stress coefficients specific to the characteristics of the species of interest would perhaps produce a more useful spatial representation and metric to test.

Moreover, it is important to note that the variable importance results reported refer to the individual direct influence of that variable on the model projection, it does not account for *interactions* between the variables or combined effects of the variables—a key tenet of SDM approaches. For example, while cloud cover has in general shown low levels of individual variable importance, Figure 6c demonstrated that SDM scenario 4 was the only ensemble projection to identify an increase in land suitability estimates from the bioclim‐only model—suggesting that the role of cloud cover (or rather the inverse) is significant in determining the ecological niche of *E*. *tirucalli*, albeit likely through interactions with other variables. Equally, despite the consistently high TSS values and lack of variability between the different SDM predictor sets studied (Table 3), the spatial distribution in the ecological niche suitability estimates is shown to vary between scenarios (Figures 5 and 6). These results suggest that despite marginal variation in TSS score or variable importance factors, the interactions between variables are important in explaining the overall projected suitability profile for individual species, and the minimum temperature of the coldest month, while important, is not exclusively the sole variable which defines the distribution of either species. Rather, it is the combination of both parameters documenting minimum temperatures, and also a measure of precipitation (both in terms of annual total amount, and/or a measure of variability in precipitation) which are important in explaining the ecophysiological controls on these species.

This being said, while the results in the spatial deviation of individual SDM scenarios from SDM 1 projections (Figures 5 and 6) suggest variation in the continuous likelihood profiles, binary cut‐off levels (Table 4) suggest that all alternative (i.e., SDMs 2–5) SDMs for *O*. *ficus indica* predict a reduction in suitable area relative to SDM 1, while *E*. *tirucalli* results suggest SDM 4 projects marginally greater levels of suitability than SDM 1 when assessed at a continent‐scale, for example. Thus, while the continuous suitability profiles may show one measure of difference between the alternative predictor scenarios, the binary levels of “suitable” versus “unsuitable” areas in absolute terms provide an alternative interpretation of the overall size of the ecological niche. Nevertheless, despite these values suggesting >15 million km^2^ of suitable area for *O*. *ficus indica* (e.g., SDM 1) across Africa, the potential yields will vary within these locations/SDM projections and hence a combination of both the continuous scale likelihood and the binary cut‐off values is useful in assessing the true scale of potential niche that could be used for growing these species.

### 4.2 Land suitability estimates

**FIGURE 7:**
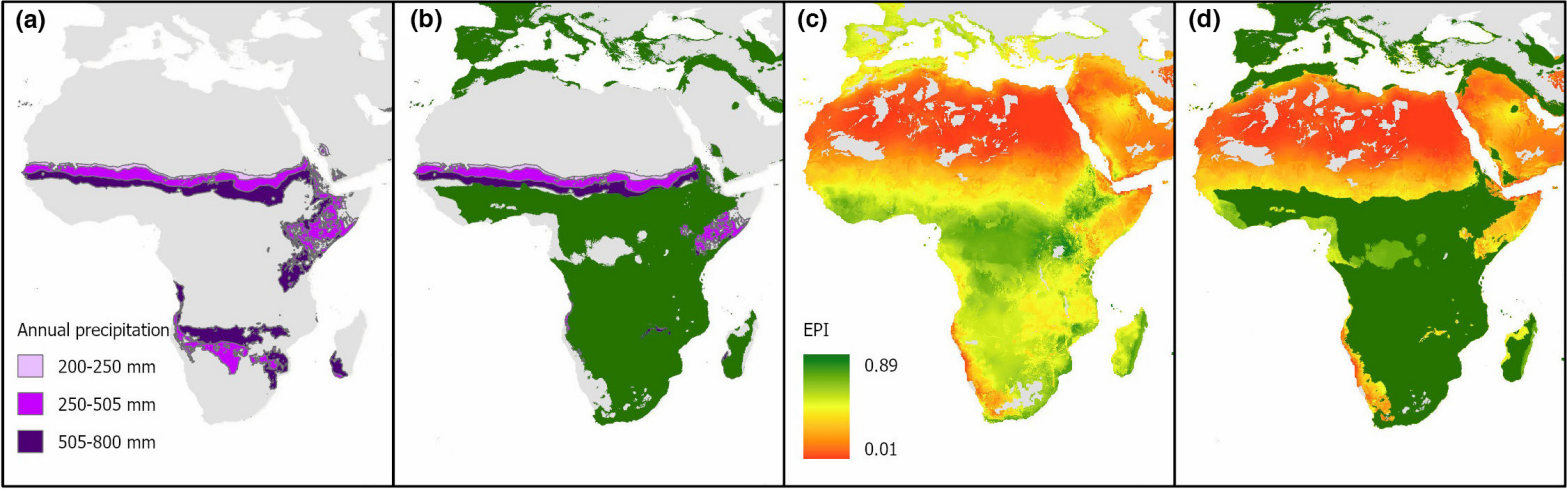
Comparison of species distribution model (SDM) 1 binary *O*. *ficus*‐*indica* projected ecological niche with existing methods from the literature: (a–b) estimates of potential area suitable for *O*. *ficus*‐*indica* growth based on the method described in Louhaichi et al. (2015) overlain with SDM 1 binary projections (this study) (c ‐ d) refined Environmental Productivity Index (EPI) for *O*. *ficus*‐*indica* as calculated in Owen et al. (2015) overlain with SDM 1 binary projections (this study)

A key advantage of the SDM approach is the capacity to produce a more refined estimate of land area that is potentially available, after taking account of protected areas and other essential land covers and uses, for cultivation of *O*. *ficus*‐*indica* and *E*. *tirucalli*. Given the overall lack of variability found between the five SDM scenarios and the equally high performance of the bioclim‐only SDM 1 model, the following section opted to only compare the results from the *O*. *ficus*‐*indica* SDM 1 model with existing methods from previous literature focusing specifically on Africa as an example region. Figure 7 presents the comparison of the land suitability estimates found in this study following SDM 1 (binary cut‐off) and the predicted suitable areas for *O*. *ficus*‐*indica* growth according to the parameters detailed in Louhaichi et al. (2015), and the adapted productivity index displayed in Owen et al. (2015).

Figure 7b overlays the results from this study onto the theoretical distribution of *O*. *ficus*‐*indica* across Africa according to the parameters detailed in Louhaichi et al. (2015) (Figure 7a), with results showing additional theoretically suitable areas in northern Africa bordering the Mediterranean, a greater region in eastern Africa, and more extensive suitability in southern Africa. SDM 1 projected distribution details a far greater suitable area than the approach taken in Louhaichi et al. (2015) since they are based on observed occurrence data rather than restricted by the common intersection of a few environmental conditions. While the models used in this study do not consider any soil‐based parameters, they have still explained over 93% of the occurrences observed with high AUC scores. When compared with the results of the productivity analysis in Owen et al. (2015), our results show a clear omission of *O*. *ficus*‐*indica* growth in central Africa where Figure 7c suggests a zone of high productivity. This is a good demonstration that our approach has taken the “competition” aspect into consideration as the EPI method suggests that *O*. *ficus*‐*indica* would grow well in central Africa, but we know through lack of occurrences in these areas that *O*. *ficus*‐*indica* is out‐competed by other plants.

Unlike the two alternative methods described above, the SDM method explored in this study is driven by the relationship with known occurrences and climatic parameters, allowing us to qualify these maps with a level of evaluative performance. As noted earlier, this suggests that c.1,500 million hectares of land are suitable for *O*. *ficus*‐*indica* and *E*. *tirucalli* growth and is of importance to initiatives looking at the potential use of CAM plant biomass as feedstock for anaerobic digestion and bioenergy, or alternative hydrolysis and VFA uses such as bioplastics, proteins. The advantage of an SDM‐based approach which incorporates the nuances and complexities of the relationships between environmental parameters and known occurrences, is that while tropical areas are theoretically identified of potential high productivity, *O*. *ficus*‐*indica* is outcompeted and occurrence data demonstrates that it is not a successful plant in these regions for reasons beyond its direct relationship with climate. This conclusion is key to identifying the most appropriate regions for exploring the potential for cultivation of CAM plants, such as *O*. *ficus*‐*indica* and *E*. *tirucalli*, as it removes any discussion regarding the removal of prime forest ecosystems in place of CAM cultivation.

### 4.3 Updated Hellmann–Eberle quotient map

**FIGURE 8:**
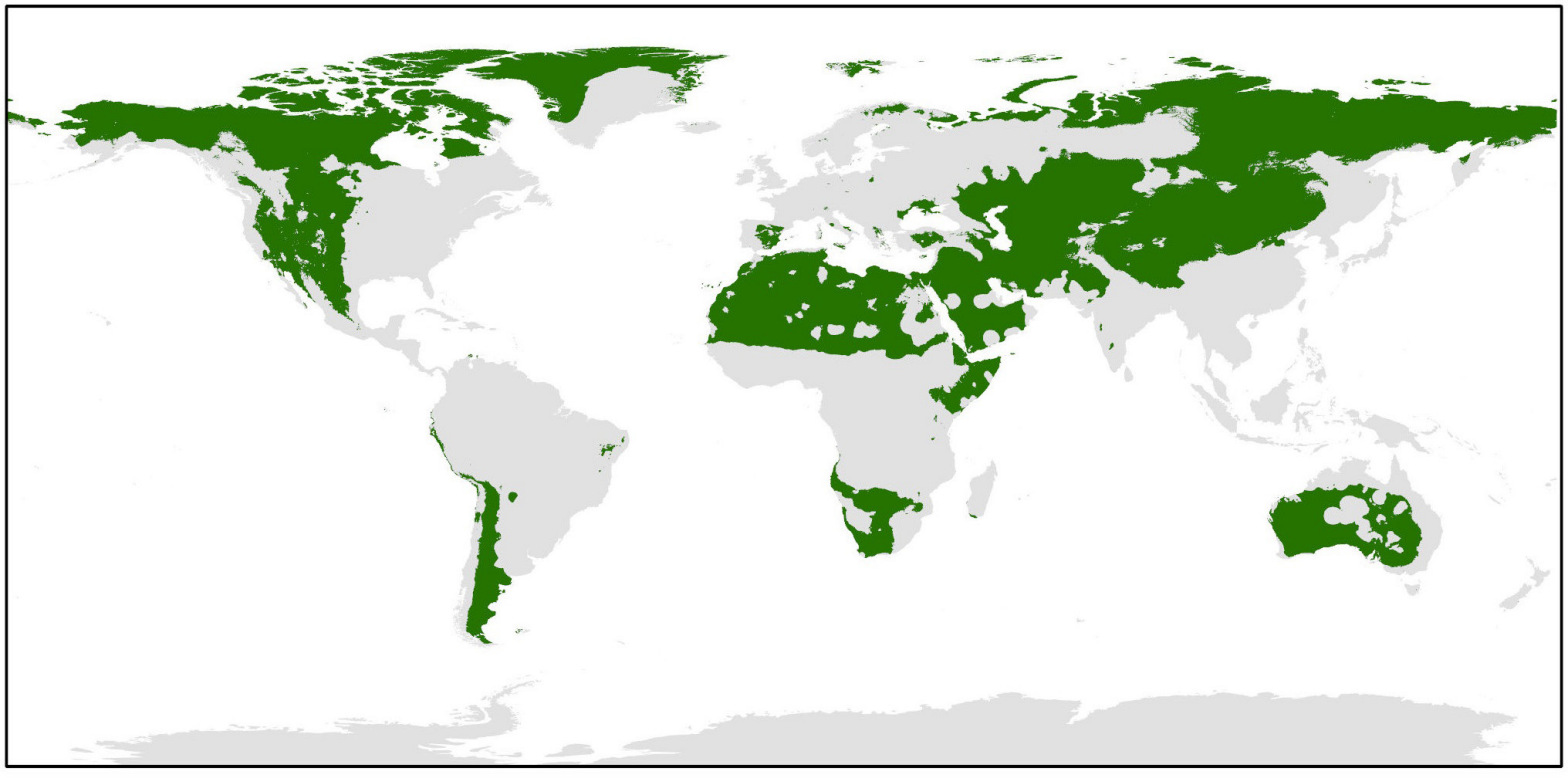
Based on the monthly precipitation values from 1960 to 2018, average annual precipitation, the Hellmann–Eberle quotient (maximum annual precipitation/minimum annual precipitation), and overall Ellenberg index were calculated. Green areas represent regions where <500 mm of rainfall coincides with Hellmann–Eberle quotient >5. Global raster of Ellenberg index as shown was used as a predictor dataset for species distribution model 2

While minimum temperatures were demonstrated as key in determining the majority of the variability in spatial distribution of the species, analysis of an updated Ellenberg index (Hellmann–Eberle quotient combined with average annual precipitation) also highlighted the importance of precipitation predictability in the distribution of succulents. As noted above, Ellenberg (1981) examined the distribution pattern of tall stem succulents in relation to climate (von Willert et al., 1992) and found that they tended to occur in areas where rainfall was low (i.e., <500 mm), but regularly received (Hellmann–Eberle quotient <5 over a long series of years) (Cowling et al., 1997). Since Ellenberg’s original study, which was based on precipitation data from 1905 to 1940, further studies have also explored the predictability of rainfall as a parameter by which to explain succulent distributions (Holtum et al., 2016; Ringelberg et al., 2020). As part of this study, an updated global Hellmann–Eberle quotient based on a longer time‐series of monthly precipitation data from 1960 to 2018 was used as a predictor parameter for the ensemble model. In addition to use in the ensemble modeling, the updated map of a revised “Ellenberg index” shown in Figure 8 provides further valuable discussion to unresolved problems regarding succulent distribution. The near absence of stem succulents from arid Australia, for example, is one particular example which has invited discussion among research groups (Holtum et al., 2016; Ringelberg et al., 2020). While Ellenberg (1981) suggested the rainfall is too unpredictable to support stem succulents in arid Australia, Ringelberg et al.’s (2020) recent ensemble model of the wider succulent biome has suggested that large parts of Australia should be climatically suitable for stem succulents; further complicating their apparent absence. Instead, Ringelberg et al. (2020) suggest that perhaps longer‐term climatic oscillations, or even historical fire conditions, may offer an alternative rationale for their absence despite favorable climatic conditions, according to their ensemble models.

By comparison, the updated Hellmann–Eberle quotient and “Ellenberg index” maps produced using a much longer period of climate data (58 years) in this study have successfully identified regions that are well‐known areas depauperate in succulents, like central Australia and large parts of Kalahari/Namib deserts. Additionally, it is highlighting other areas that agree well with observation—parts of the Arabian Peninsula, Horn of Africa, Saharan desert, and in South America the Atacama. This updated visualization based on a longer time series than previously studied suggests that perhaps high variability in annual precipitation levels over the long term is key to explaining succulent absence, such as the lack of endemic terrestrial species with CAM in arid Australia.

## 5 CONCLUSIONS

In comparison with existing methods of land suitability estimation for these species, this study has taken an a posteriori modeling approach using SDMs and known occurrences to extrapolate wider areas of potential suitability for cultivation of these species. In doing so, it has allowed us to qualify the models of suitability estimates with a level of evaluative performance, incorporates the nuances and complexities of relationships between environmental parameters and known occurrences, and produce a more refined estimate of land area that is potentially available for cultivation of *O*. *ficus*‐*indica* and *E*. *tirucalli* when considered alongside existing land uses and primary ecosystems. The high model performance metrics of SDMs made using successfully invasive distribution‐unlimited species gives us confidence that most of the fundamental niche of *O*. *ficus*‐*indica* and *E*. *tirucalli* can be explained by the models produced in this study, and given the negligible variability between the different scenarios, there is no benefit in expanding model complexity and increasing the potential for over‐fitting by including additional environmental predictors. While the minimum temperature of the coldest month was found to be the key variable of importance in determining the spatial variability of *O*. *ficus*‐*indica* and *E*. *tirucalli*, these results are based on the individual performance of each parameter as opposed to combined effects and nonlinearities between the environmental predictors. An updated global map of Hellmann–Eberle quotient based on a much longer period of climate data (ca. 60 years), supports the ideas of Ellenberg (1981) that long‐term precipitation variability is also a key variable in determining CAM plant distribution, and in certain regions can explain stem succulent absence.

## AUTHOR CONTRIBUTION

**Catherine E. Buckland:** Conceptualization (lead); Data curation (lead); Formal analysis (lead); Investigation (lead); Methodology (lead); Writing – original draft (lead); Writing – review & editing (lead). **Andrew J.A.C. Smith:** Formal analysis (supporting); Funding acquisition (equal); Investigation (supporting); Supervision (supporting); Writing – review & editing (supporting). **David S. G. Thomas:** Funding acquisition (equal); Investigation (supporting); Supervision (supporting).

## CONFLICT OF INTEREST

The authors declare no competing interests.

## Supporting information

Supplementary Material

## Data Availability

The authors confirm that the results supporting the findings of this study are available within the article and its supplementary materials. Raw data and code (R script) to reproduce the results are available at Dryad: https://doi.org/10.5061/dryad.wwpzgmsmt.
