# Supplementary material for: A comparison in species distribution model performance of succulents using key species and subsets of environmental predictors
Source: Ecol Evol. 2022 Jun 6;12(6):e8981. doi: 10.1002/ece3.8981 (PMC9170539; doi:10.1002/ece3.8981)
Supplement: Supplementary file 1 — Supplementary Material [file ECE3-12-e8981-s001.docx]

***Supplementary Information***

**Appendix A: Predictor datasets**

*Bioclim variables*


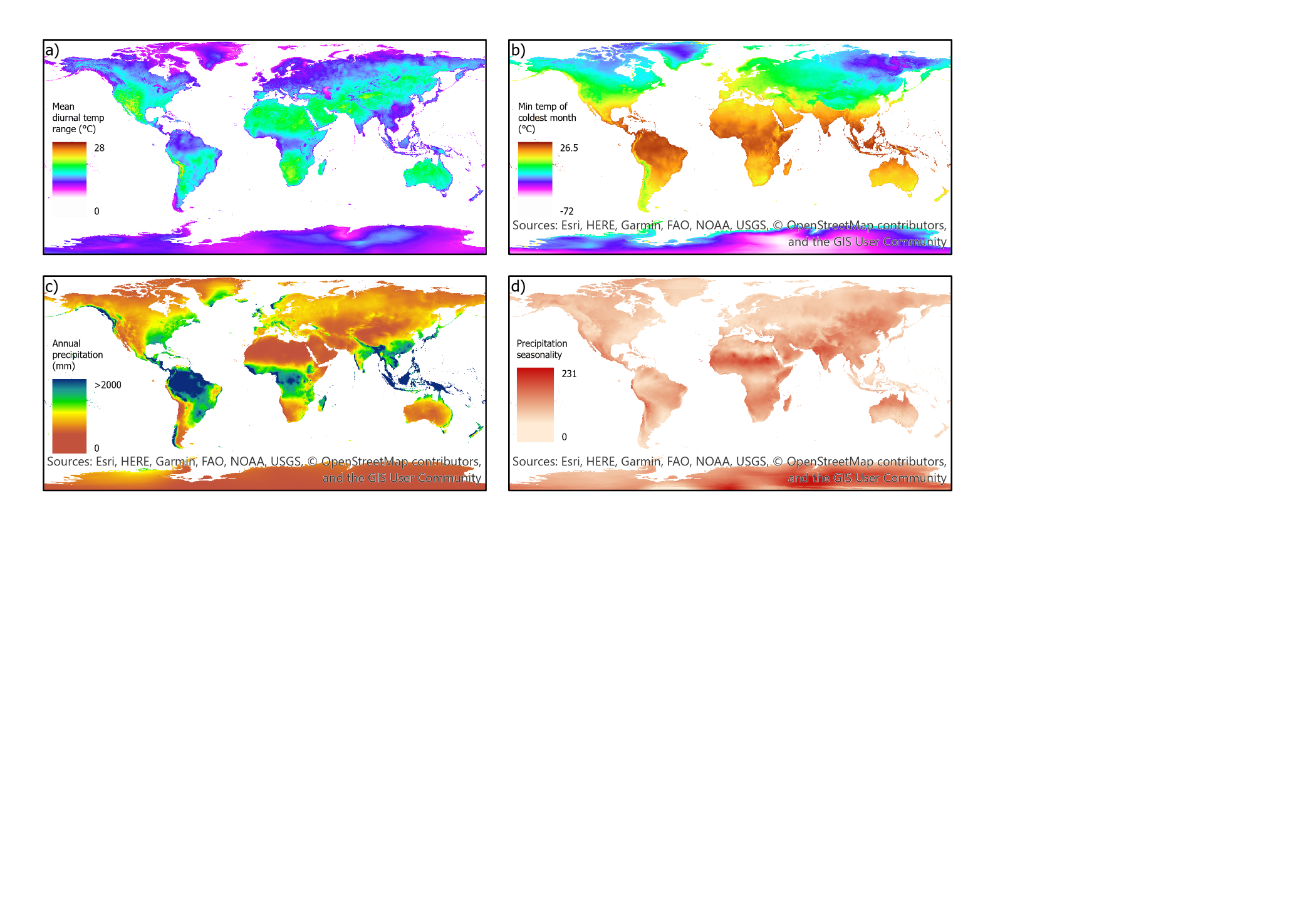
Bioclim rasters were downloaded from the WorldClim 2 (Fick and Hijmans, 2017) dataset at 2.5 min spatial resolution and are based on the climate data for 1970-2000. Based on an existing understanding of the main climatic variables considered to limit the spatial distribution of CAM species such as *Opuntia ficus-indica* and *Euphorbia tirucalli*, four bioclim variables were selected for inclusion in all of the SDM scenarios (Figure S1). Pearson’s Correlation coefficient results did not show significant covariance between these parameters (Figure S2 & Table S1).

**Figure S1.** Bioclim parameters used as predictor datasets for SDM scenarios 1-5. (a) Mean diurnal temperature range (°C), (b) Minimum temperature of the coldest month (°C), (c) Annual precipitation (mm), (d) Precipitation seasonality (coefficient of variation).

*Pearson’s Correlation coefficient*


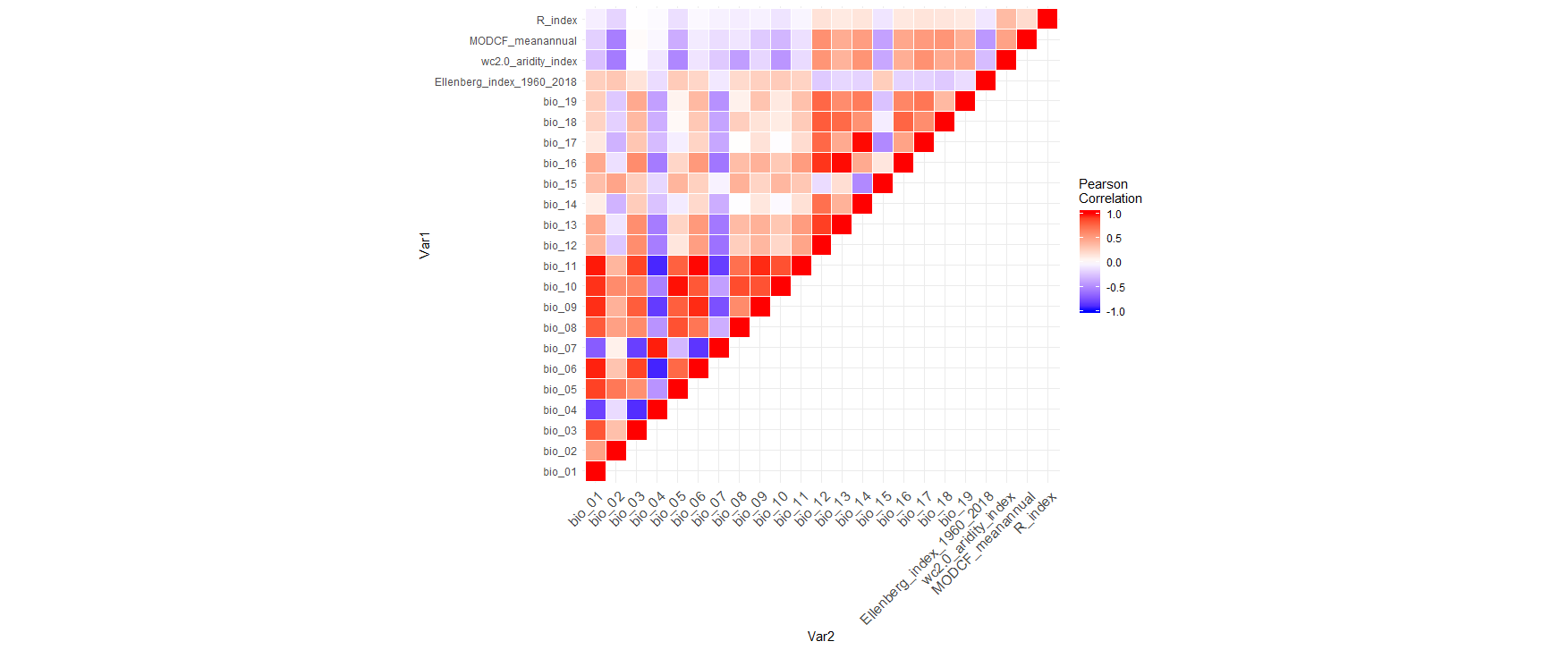

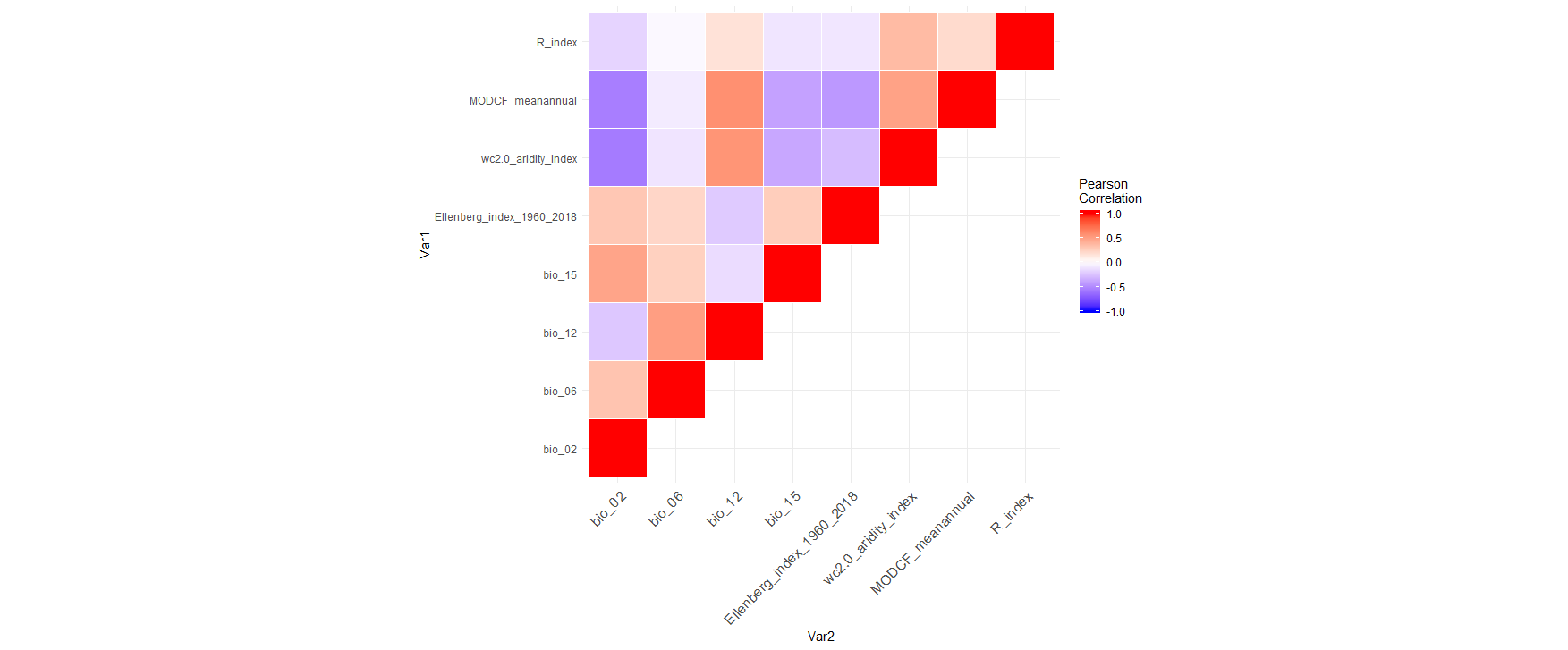


**Figure S2.** *Left:* Pairwise spatial correlation (Pearson’s *r*) results between all climatic and environmental indices considered. *Right:* Final selection of the four bioclim variables and environmental variables used across SDM scenarios 1-5.

**Table S1.** Pairwise spatial correlation (Pearson’s *r*) between climatic and environmental parameters used across SDM scenarios 1-5.

|  | **Bio 2** | **Bio 6** | **Bio 12** | **Bio 15** | **Hellmann-Eberle quotient** | **Aridity index** | **Cloud cover** | **R index** |
| --- | --- | --- | --- | --- | --- | --- | --- | --- |
| **Bio 2** |  |  |  |  |  |  |  |  |
| **Bio 6** | 0.31 |  |  |  |  |  |  |  |
| **Bio 12** | -0.24 | 0.50 |  |  |  |  |  |  |
| **Bio 15** | 0.47 | 0.24 | -0.15 |  |  |  |  |  |
| **Hellmann-Eberle quotient** | 0.29 | 0.21 | -0.23 | 0.26 |  |  |  |  |
| **Aridity index** | -0.57 | -0.11 | 0.55 | -0.38 | -0.29 |  |  |  |
| **Cloud cover** | -0.56 | -0.09 | 0.57 | -0.40 | -0.45 | 0.48 |  |  |
| **R index** | -0.18 | -0.03 | 0.15 | -0.11 | -0.11 | 0.36 | 0.19 |  |

**Appendix B: Occurrence datasets**

*Opuntia ficus-indica* and *Euphorbia tirucalli* occurrence datasets were produced from the GBIF repository before being cleaned following the method described in Zizka et al. (2019) to clean records for geo-referencing, dating imprecisions and data entry errors (Figures S3 & S4).


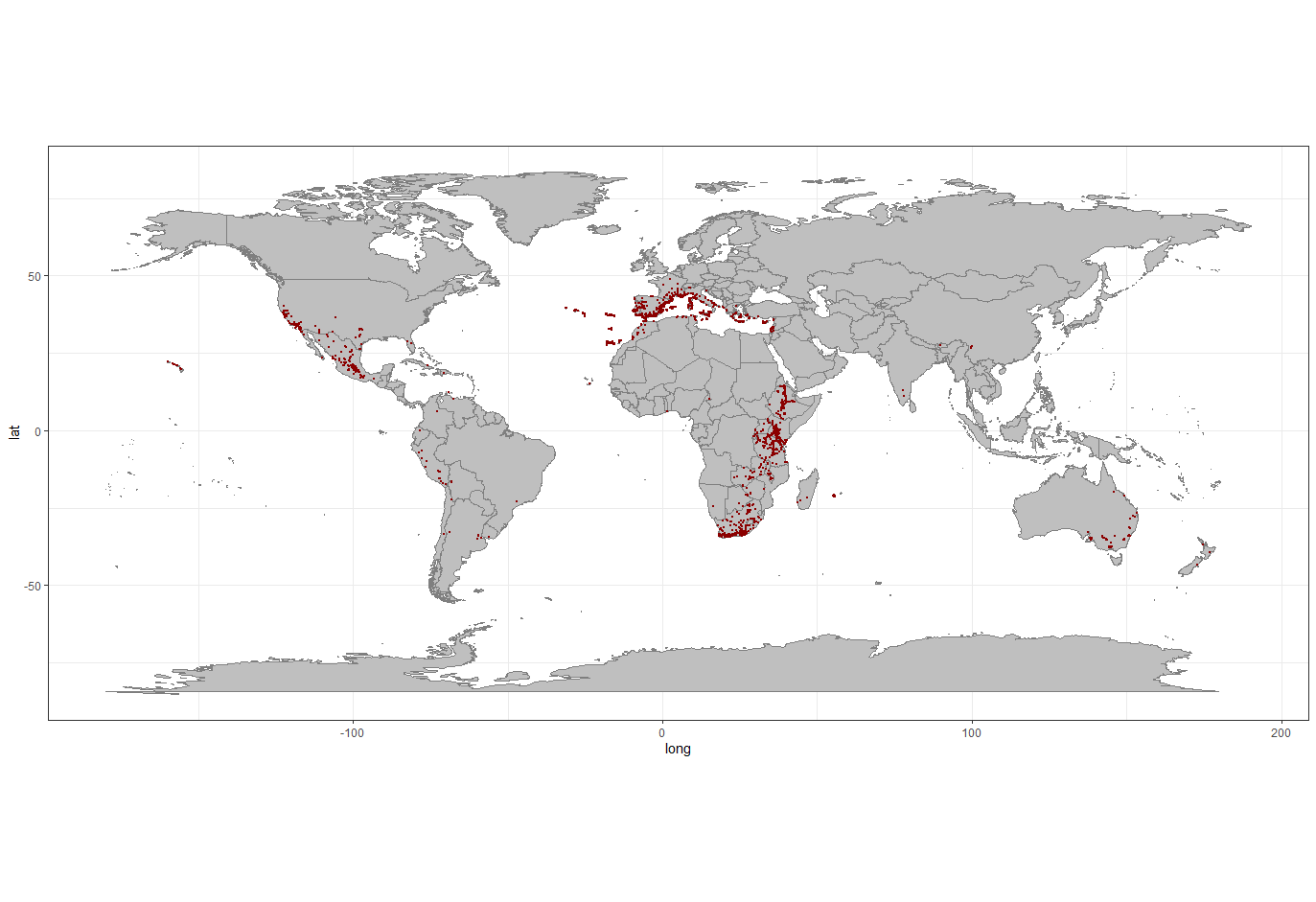

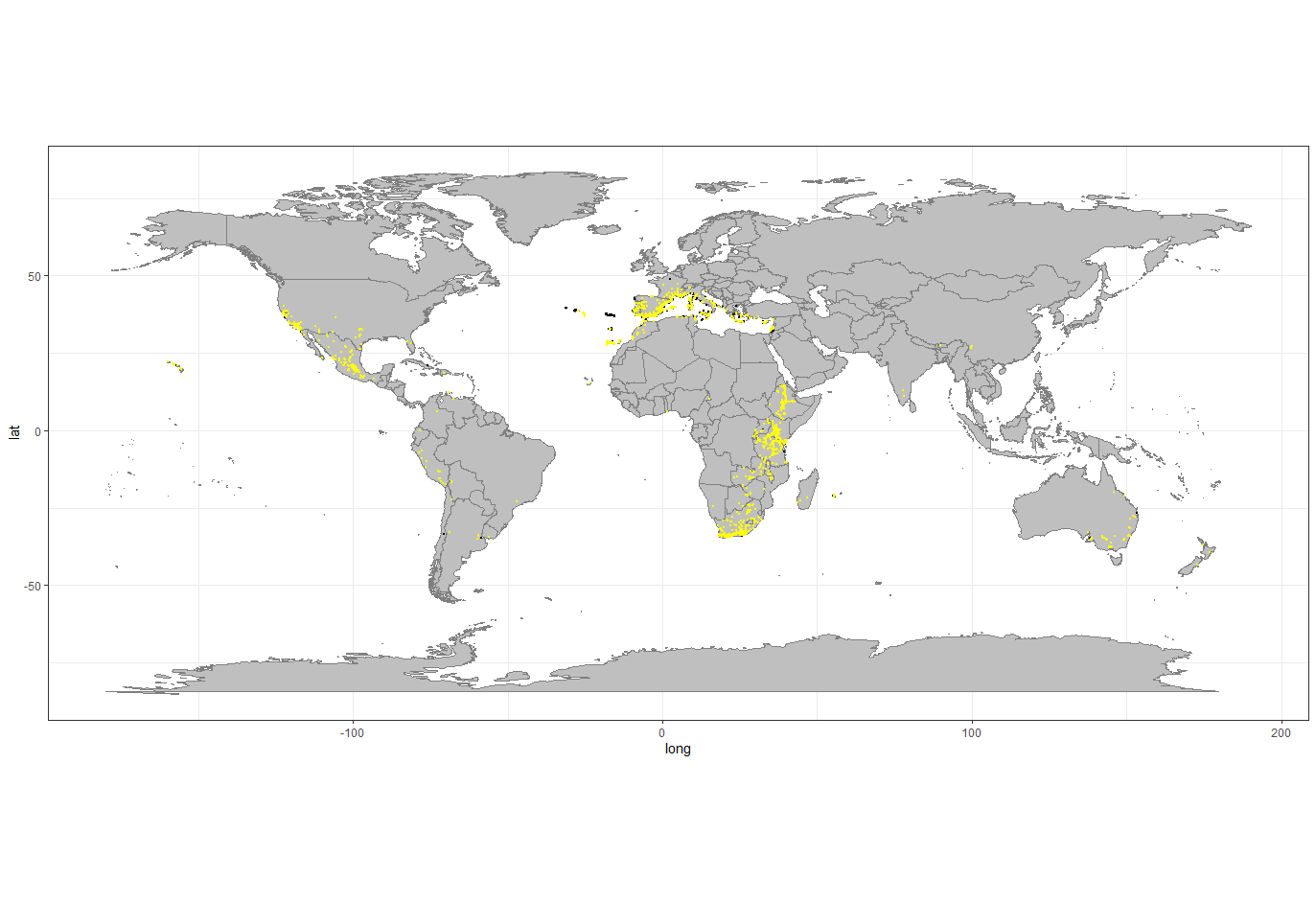

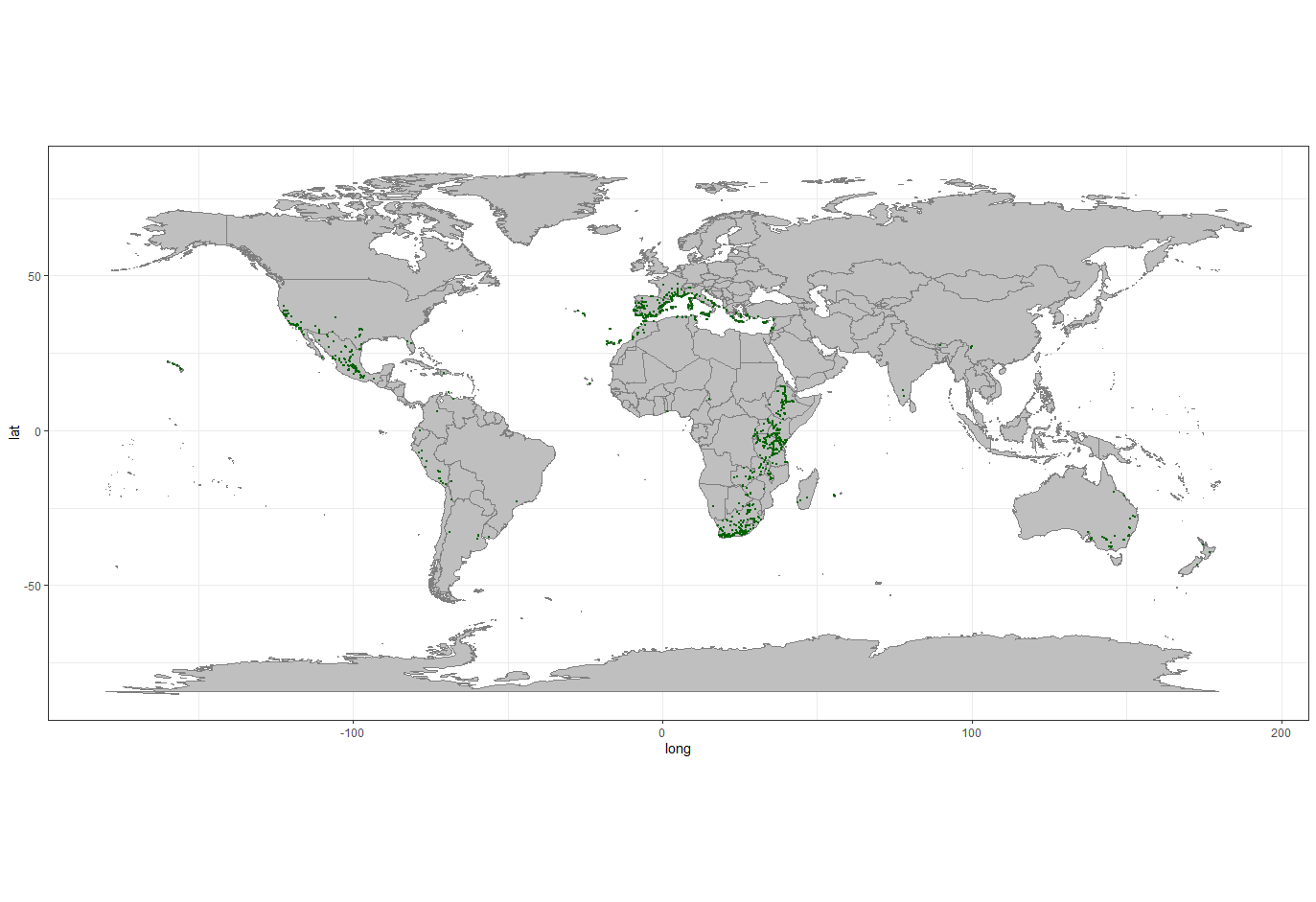


**Figure S3.** (a) All *O. ficus-indica* occurrences based on downloaded from the GBIF dataset (09/06/2020), (b) Yellow – cleaned *O. ficus-indica* occurrences, black – flagged *O. ficus-indica* occurrences, (c) Final *O. ficus-indica* occurrences selected after spatial bias analysis completed.


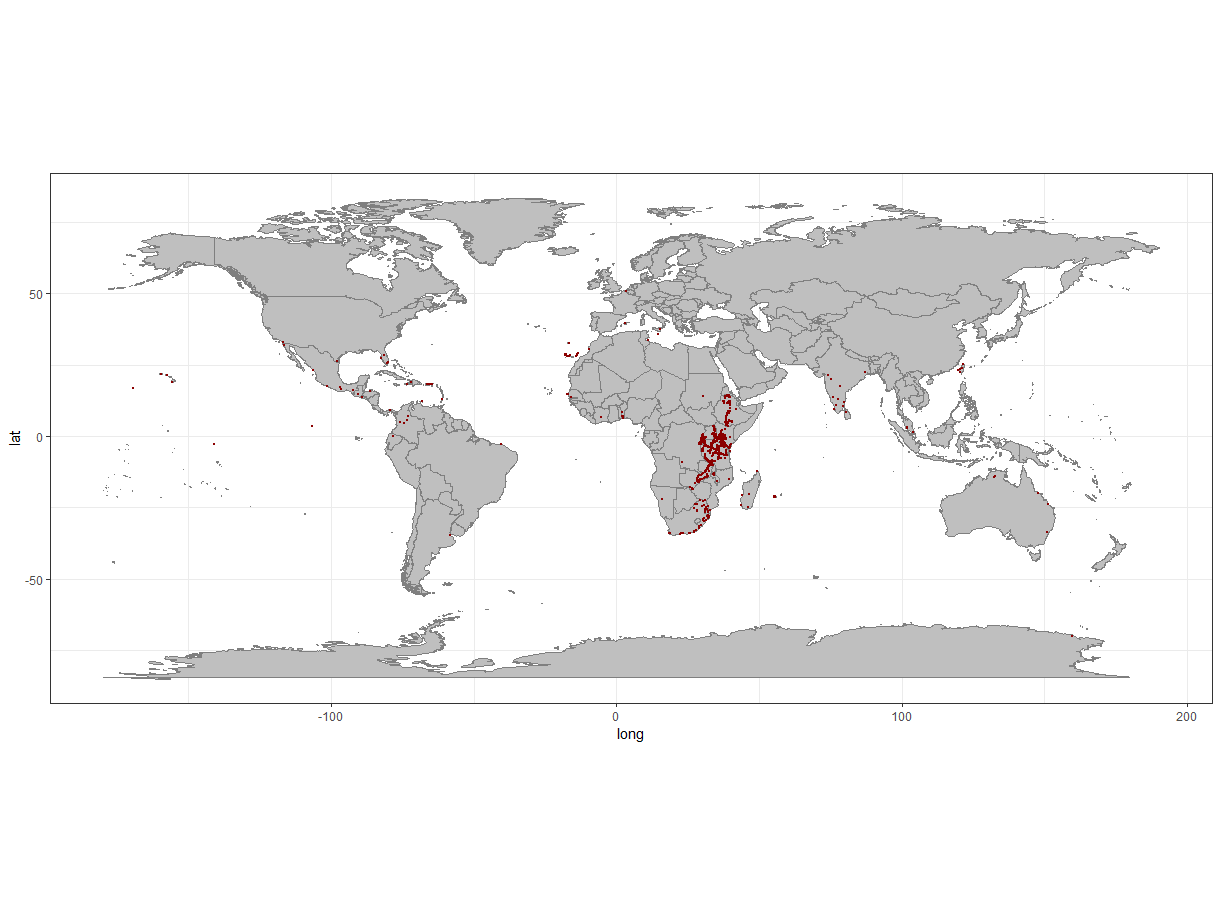

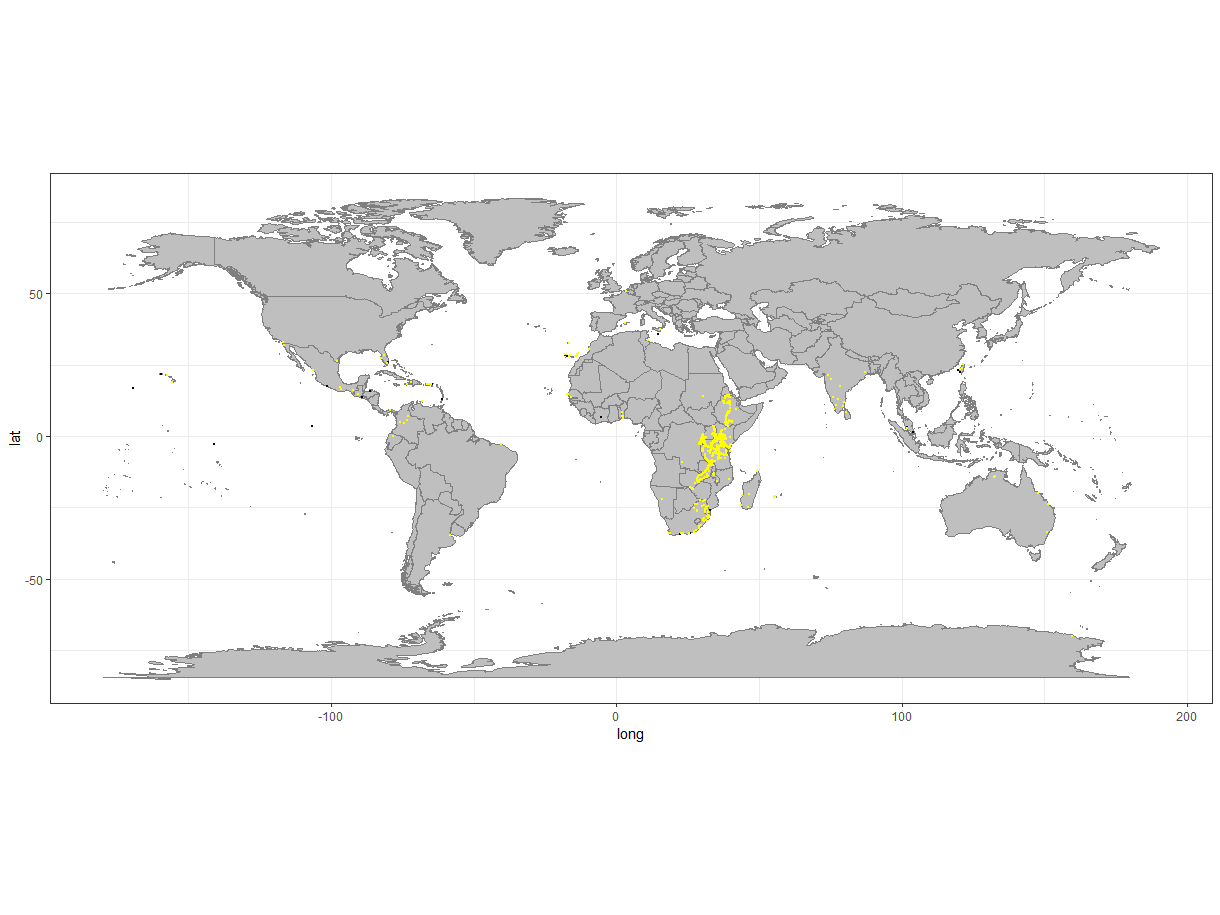

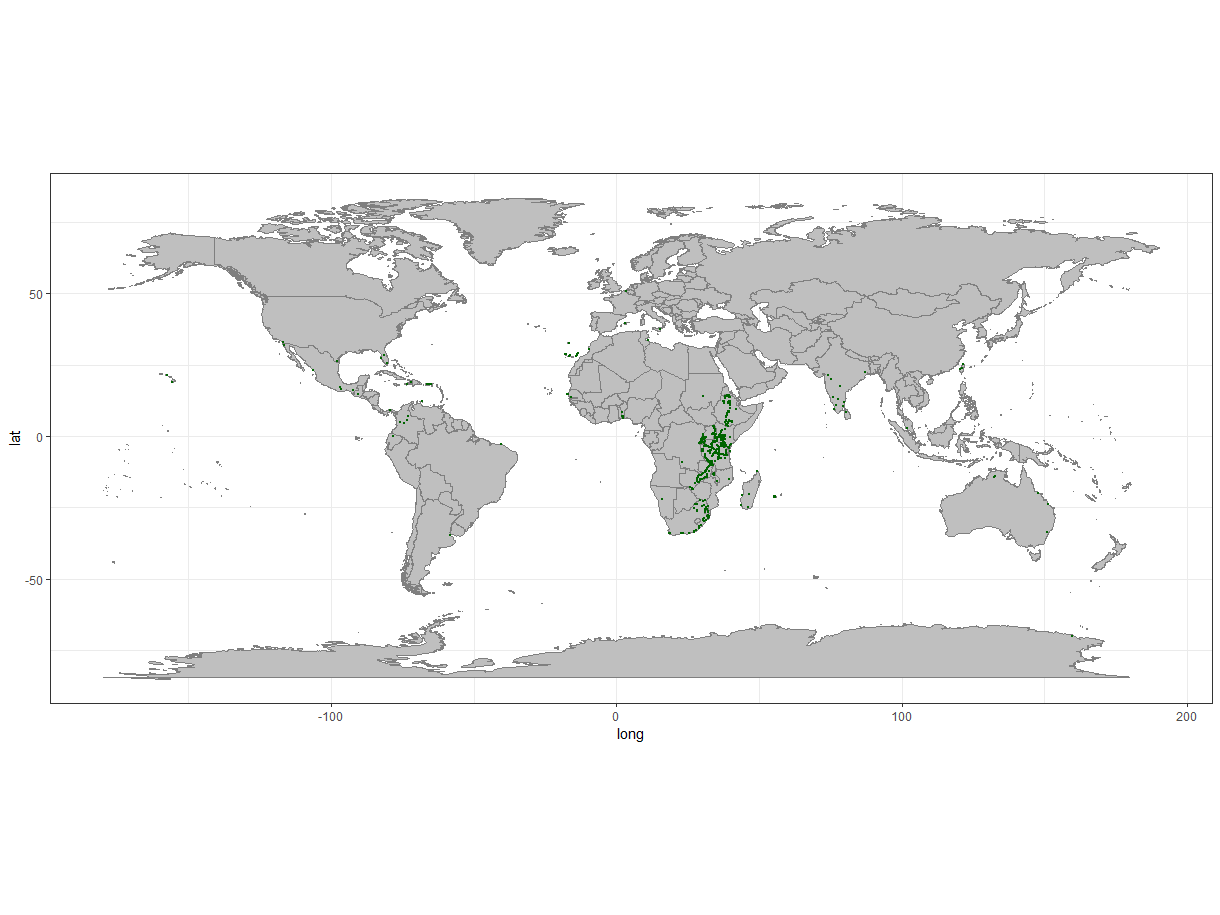


**Figure S4.** (a) All *E. tirucalli* occurrences based on downloaded from the GBIF dataset (09/06/2020), (b) Yellow – cleaned *E. tirucalli* occurrences, black – flagged *E. tirucalli* occurrences, (c) Final *E. tirucalli* occurrences selected after spatial bias analysis completed.

**Appendix C: Individual model performance metrics**

Individual model performance metrics for *Opuntia ficus-indica* and *Euphorbia tirucalli* by algorithm (Random Forest, Generalised Boosting Model), repeat (Run 1-10) and pseudo-absence (PA 1-5).

| ***Opuntia ficus-indica*** | | **ROC** | | | | | **TSS** | | | | |
| --- | --- | --- | --- | --- | --- | --- | --- | --- | --- | --- | --- |
| **SDM 1** | | **PA1** | **PA2** | **PA3** | **PA4** | **PA5** | **PA1** | **PA2** | **PA3** | **PA4** | **PA5** |
| **RF** | RUN1 | 0.993 | 0.994 | 0.992 | 0.995 | 0.994 | 0.907 | 0.925 | 0.898 | 0.924 | 0.917 |
|  | RUN2 | 0.993 | 0.994 | 0.992 | 0.995 | 0.995 | 0.91 | 0.921 | 0.908 | 0.932 | 0.92 |
|  | RUN3 | 0.992 | 0.996 | 0.995 | 0.996 | 0.995 | 0.915 | 0.932 | 0.927 | 0.93 | 0.924 |
|  | RUN4 | 0.994 | 0.995 | 0.994 | 0.994 | 0.995 | 0.916 | 0.926 | 0.924 | 0.92 | 0.917 |
|  | RUN5 | 0.993 | 0.995 | 0.994 | 0.993 | 0.994 | 0.912 | 0.921 | 0.914 | 0.914 | 0.918 |
|  | RUN6 | 0.994 | 0.994 | 0.994 | 0.994 | 0.996 | 0.914 | 0.922 | 0.91 | 0.914 | 0.924 |
|  | RUN7 | 0.992 | 0.994 | 0.994 | 0.996 | 0.995 | 0.903 | 0.917 | 0.914 | 0.933 | 0.93 |
|  | RUN8 | 0.994 | 0.994 | 0.993 | 0.996 | 0.996 | 0.921 | 0.906 | 0.913 | 0.927 | 0.935 |
|  | RUN9 | 0.994 | 0.994 | 0.995 | 0.994 | 0.994 | 0.911 | 0.913 | 0.917 | 0.927 | 0.92 |
|  | RUN10 | 0.99 | 0.994 | 0.993 | 0.995 | 0.994 | 0.904 | 0.918 | 0.914 | 0.914 | 0.926 |
| **GBM** | RUN1 | 0.991 | 0.994 | 0.987 | 0.993 | 0.992 | 0.894 | 0.926 | 0.885 | 0.903 | 0.904 |
|  | RUN2 | 0.989 | 0.991 | 0.99 | 0.992 | 0.993 | 0.893 | 0.905 | 0.891 | 0.912 | 0.915 |
|  | RUN3 | 0.99 | 0.995 | 0.993 | 0.992 | 0.992 | 0.898 | 0.921 | 0.908 | 0.911 | 0.904 |
|  | RUN4 | 0.991 | 0.993 | 0.989 | 0.991 | 0.991 | 0.902 | 0.906 | 0.899 | 0.889 | 0.895 |
|  | RUN5 | 0.991 | 0.993 | 0.992 | 0.991 | 0.992 | 0.902 | 0.909 | 0.91 | 0.896 | 0.898 |
|  | RUN6 | 0.991 | 0.992 | 0.99 | 0.991 | 0.994 | 0.888 | 0.912 | 0.897 | 0.893 | 0.911 |
|  | RUN7 | 0.989 | 0.992 | 0.993 | 0.992 | 0.993 | 0.885 | 0.903 | 0.911 | 0.903 | 0.912 |
|  | RUN8 | 0.992 | 0.992 | 0.991 | 0.993 | 0.994 | 0.903 | 0.898 | 0.899 | 0.905 | 0.916 |
|  | RUN9 | 0.99 | 0.992 | 0.992 | 0.99 | 0.991 | 0.904 | 0.906 | 0.905 | 0.904 | 0.903 |
|  | RUN10 | 0.988 | 0.993 | 0.989 | 0.991 | 0.992 | 0.887 | 0.908 | 0.896 | 0.904 | 0.913 |
| **SDM 2** | | **ROC** | | | | | **TSS** | | | | |
| **RF** | RUN1 | 0.992 | 0.989 | 0.991 | 0.991 | 0.991 | 0.908 | 0.894 | 0.892 | 0.914 | 0.913 |
|  | RUN2 | 0.991 | 0.992 | 0.988 | 0.992 | 0.991 | 0.907 | 0.905 | 0.889 | 0.906 | 0.904 |
|  | RUN3 | 0.993 | 0.988 | 0.991 | 0.992 | 0.991 | 0.913 | 0.884 | 0.907 | 0.913 | 0.913 |
|  | RUN4 | 0.989 | 0.989 | 0.99 | 0.99 | 0.992 | 0.903 | 0.899 | 0.9 | 0.906 | 0.909 |
|  | RUN5 | 0.99 | 0.989 | 0.988 | 0.991 | 0.991 | 0.897 | 0.892 | 0.891 | 0.906 | 0.914 |
|  | RUN6 | 0.992 | 0.993 | 0.989 | 0.988 | 0.991 | 0.909 | 0.905 | 0.889 | 0.894 | 0.907 |
|  | RUN7 | 0.992 | 0.992 | 0.99 | 0.99 | 0.989 | 0.914 | 0.91 | 0.901 | 0.901 | 0.899 |
|  | RUN8 | 0.991 | 0.99 | 0.991 | 0.989 | 0.991 | 0.904 | 0.9 | 0.91 | 0.904 | 0.904 |
|  | RUN9 | 0.992 | 0.991 | 0.992 | 0.993 | 0.99 | 0.905 | 0.903 | 0.905 | 0.914 | 0.898 |
|  | RUN10 | 0.993 | 0.988 | 0.99 | 0.989 | 0.99 | 0.912 | 0.892 | 0.902 | 0.891 | 0.902 |
| **GBM** | RUN1 | 0.986 | 0.982 | 0.986 | 0.986 | 0.985 | 0.874 | 0.864 | 0.869 | 0.883 | 0.877 |
|  | RUN2 | 0.984 | 0.987 | 0.983 | 0.986 | 0.983 | 0.873 | 0.889 | 0.865 | 0.874 | 0.875 |
|  | RUN3 | 0.988 | 0.984 | 0.985 | 0.986 | 0.986 | 0.895 | 0.861 | 0.874 | 0.873 | 0.886 |
|  | RUN4 | 0.982 | 0.983 | 0.984 | 0.984 | 0.986 | 0.875 | 0.868 | 0.867 | 0.867 | 0.879 |
|  | RUN5 | 0.985 | 0.986 | 0.984 | 0.986 | 0.986 | 0.867 | 0.872 | 0.861 | 0.881 | 0.899 |
|  | RUN6 | 0.987 | 0.988 | 0.985 | 0.983 | 0.987 | 0.884 | 0.887 | 0.873 | 0.865 | 0.879 |
|  | RUN7 | 0.988 | 0.987 | 0.984 | 0.986 | 0.984 | 0.889 | 0.886 | 0.863 | 0.882 | 0.873 |
|  | RUN8 | 0.986 | 0.985 | 0.986 | 0.985 | 0.986 | 0.875 | 0.882 | 0.882 | 0.868 | 0.874 |
|  | RUN9 | 0.988 | 0.985 | 0.987 | 0.987 | 0.986 | 0.879 | 0.881 | 0.877 | 0.879 | 0.877 |
|  | RUN10 | 0.987 | 0.982 | 0.984 | 0.982 | 0.985 | 0.879 | 0.85 | 0.87 | 0.857 | 0.889 |
| **SDM 3** | | **ROC** | | | | | **TSS** | | | | |
| **RF** | RUN1 | 0.992 | 0.99 | 0.994 | 0.991 | 0.99 | 0.908 | 0.892 | 0.913 | 0.898 | 0.903 |
|  | RUN2 | 0.989 | 0.991 | 0.991 | 0.991 | 0.992 | 0.891 | 0.9 | 0.912 | 0.899 | 0.91 |
|  | RUN3 | 0.992 | 0.99 | 0.988 | 0.993 | 0.991 | 0.908 | 0.902 | 0.886 | 0.914 | 0.904 |
|  | RUN4 | 0.991 | 0.991 | 0.989 | 0.993 | 0.991 | 0.905 | 0.903 | 0.904 | 0.912 | 0.911 |
|  | RUN5 | 0.99 | 0.989 | 0.989 | 0.991 | 0.99 | 0.91 | 0.891 | 0.892 | 0.907 | 0.9 |
|  | RUN6 | 0.991 | 0.989 | 0.992 | 0.99 | 0.992 | 0.91 | 0.886 | 0.908 | 0.898 | 0.9 |
|  | RUN7 | 0.994 | 0.992 | 0.992 | 0.992 | 0.99 | 0.922 | 0.906 | 0.917 | 0.912 | 0.911 |
|  | RUN8 | 0.991 | 0.991 | 0.99 | 0.99 | 0.991 | 0.917 | 0.908 | 0.89 | 0.898 | 0.903 |
|  | RUN9 | 0.99 | 0.991 | 0.991 | 0.991 | 0.989 | 0.891 | 0.9 | 0.906 | 0.909 | 0.905 |
|  | RUN10 | 0.991 | 0.992 | 0.991 | 0.992 | 0.99 | 0.901 | 0.898 | 0.9 | 0.903 | 0.898 |
| **GBM** | RUN1 | 0.987 | 0.986 | 0.988 | 0.987 | 0.986 | 0.89 | 0.869 | 0.882 | 0.876 | 0.88 |
|  | RUN2 | 0.984 | 0.988 | 0.985 | 0.986 | 0.987 | 0.874 | 0.873 | 0.874 | 0.864 | 0.882 |
|  | RUN3 | 0.986 | 0.985 | 0.984 | 0.988 | 0.988 | 0.885 | 0.887 | 0.863 | 0.89 | 0.878 |
|  | RUN4 | 0.987 | 0.987 | 0.986 | 0.988 | 0.986 | 0.877 | 0.87 | 0.883 | 0.885 | 0.875 |
|  | RUN5 | 0.987 | 0.986 | 0.985 | 0.986 | 0.986 | 0.88 | 0.87 | 0.861 | 0.876 | 0.878 |
|  | RUN6 | 0.987 | 0.985 | 0.987 | 0.985 | 0.989 | 0.881 | 0.865 | 0.88 | 0.877 | 0.884 |
|  | RUN7 | 0.99 | 0.987 | 0.988 | 0.988 | 0.986 | 0.9 | 0.873 | 0.89 | 0.891 | 0.877 |
|  | RUN8 | 0.988 | 0.987 | 0.985 | 0.985 | 0.988 | 0.886 | 0.89 | 0.869 | 0.869 | 0.88 |
|  | RUN9 | 0.984 | 0.987 | 0.988 | 0.986 | 0.984 | 0.873 | 0.869 | 0.888 | 0.879 | 0.868 |
|  | RUN10 | 0.987 | 0.987 | 0.986 | 0.989 | 0.985 | 0.868 | 0.877 | 0.88 | 0.88 | 0.876 |
| **SDM 4** | | **ROC** | | | | | **TSS** | | | | |
| **RF** | RUN1 | 0.991 | 0.995 | 0.992 | 0.992 | 0.994 | 0.903 | 0.934 | 0.907 | 0.899 | 0.914 |
|  | RUN2 | 0.992 | 0.994 | 0.993 | 0.994 | 0.993 | 0.903 | 0.924 | 0.911 | 0.916 | 0.922 |
|  | RUN3 | 0.994 | 0.994 | 0.993 | 0.992 | 0.993 | 0.909 | 0.926 | 0.914 | 0.909 | 0.914 |
|  | RUN4 | 0.995 | 0.994 | 0.991 | 0.994 | 0.992 | 0.927 | 0.924 | 0.915 | 0.907 | 0.903 |
|  | RUN5 | 0.994 | 0.996 | 0.992 | 0.993 | 0.994 | 0.916 | 0.942 | 0.914 | 0.914 | 0.915 |
|  | RUN6 | 0.992 | 0.994 | 0.99 | 0.993 | 0.993 | 0.907 | 0.924 | 0.896 | 0.904 | 0.916 |
|  | RUN7 | 0.992 | 0.995 | 0.993 | 0.99 | 0.994 | 0.915 | 0.93 | 0.907 | 0.907 | 0.912 |
|  | RUN8 | 0.993 | 0.994 | 0.992 | 0.994 | 0.994 | 0.917 | 0.922 | 0.909 | 0.922 | 0.912 |
|  | RUN9 | 0.992 | 0.994 | 0.994 | 0.995 | 0.993 | 0.91 | 0.928 | 0.914 | 0.929 | 0.911 |
|  | RUN10 | 0.995 | 0.994 | 0.993 | 0.992 | 0.989 | 0.922 | 0.926 | 0.928 | 0.913 | 0.899 |
| **GBM** | RUN1 | 0.989 | 0.992 | 0.99 | 0.988 | 0.99 | 0.885 | 0.911 | 0.888 | 0.894 | 0.892 |
|  | RUN2 | 0.989 | 0.991 | 0.99 | 0.99 | 0.989 | 0.881 | 0.904 | 0.892 | 0.898 | 0.895 |
|  | RUN3 | 0.991 | 0.992 | 0.99 | 0.989 | 0.99 | 0.891 | 0.906 | 0.889 | 0.891 | 0.893 |
|  | RUN4 | 0.992 | 0.992 | 0.99 | 0.992 | 0.99 | 0.904 | 0.913 | 0.899 | 0.901 | 0.891 |
|  | RUN5 | 0.992 | 0.994 | 0.991 | 0.991 | 0.99 | 0.904 | 0.927 | 0.894 | 0.9 | 0.891 |
|  | RUN6 | 0.989 | 0.993 | 0.988 | 0.99 | 0.99 | 0.887 | 0.908 | 0.887 | 0.893 | 0.89 |
|  | RUN7 | 0.991 | 0.994 | 0.99 | 0.988 | 0.991 | 0.894 | 0.921 | 0.884 | 0.884 | 0.882 |
|  | RUN8 | 0.99 | 0.992 | 0.99 | 0.991 | 0.991 | 0.893 | 0.9 | 0.895 | 0.902 | 0.894 |
|  | RUN9 | 0.989 | 0.992 | 0.991 | 0.992 | 0.99 | 0.885 | 0.912 | 0.892 | 0.904 | 0.889 |
|  | RUN10 | 0.993 | 0.992 | 0.99 | 0.991 | 0.987 | 0.908 | 0.913 | 0.909 | 0.908 | 0.873 |
| **SDM 5** | | **ROC** | | | | | **TSS** | | | | |
| **RF** | RUN1 | 0.993 | 0.992 | 0.989 | 0.993 | 0.991 | 0.912 | 0.91 | 0.893 | 0.924 | 0.907 |
|  | RUN2 | 0.992 | 0.992 | 0.991 | 0.993 | 0.993 | 0.904 | 0.915 | 0.912 | 0.914 | 0.904 |
|  | RUN3 | 0.992 | 0.988 | 0.99 | 0.99 | 0.991 | 0.91 | 0.897 | 0.904 | 0.902 | 0.909 |
|  | RUN4 | 0.992 | 0.991 | 0.992 | 0.994 | 0.991 | 0.912 | 0.913 | 0.904 | 0.915 | 0.907 |
|  | RUN5 | 0.991 | 0.992 | 0.99 | 0.989 | 0.991 | 0.91 | 0.913 | 0.904 | 0.894 | 0.904 |
|  | RUN6 | 0.992 | 0.992 | 0.993 | 0.994 | 0.993 | 0.907 | 0.919 | 0.921 | 0.93 | 0.911 |
|  | RUN7 | 0.992 | 0.991 | 0.992 | 0.988 | 0.991 | 0.914 | 0.918 | 0.914 | 0.889 | 0.903 |
|  | RUN8 | 0.992 | 0.99 | 0.991 | 0.99 | 0.992 | 0.91 | 0.899 | 0.911 | 0.9 | 0.912 |
|  | RUN9 | 0.993 | 0.989 | 0.99 | 0.991 | 0.992 | 0.921 | 0.899 | 0.901 | 0.912 | 0.901 |
|  | RUN10 | 0.992 | 0.991 | 0.99 | 0.991 | 0.991 | 0.907 | 0.906 | 0.908 | 0.907 | 0.901 |
| **GBM** | RUN1 | 0.988 | 0.987 | 0.985 | 0.989 | 0.987 | 0.876 | 0.884 | 0.865 | 0.893 | 0.878 |
|  | RUN2 | 0.987 | 0.987 | 0.989 | 0.989 | 0.988 | 0.872 | 0.882 | 0.889 | 0.886 | 0.878 |
|  | RUN3 | 0.988 | 0.985 | 0.985 | 0.985 | 0.988 | 0.879 | 0.87 | 0.882 | 0.881 | 0.885 |
|  | RUN4 | 0.987 | 0.986 | 0.988 | 0.99 | 0.985 | 0.874 | 0.88 | 0.884 | 0.889 | 0.871 |
|  | RUN5 | 0.986 | 0.988 | 0.986 | 0.984 | 0.987 | 0.88 | 0.88 | 0.874 | 0.875 | 0.878 |
|  | RUN6 | 0.985 | 0.988 | 0.989 | 0.99 | 0.989 | 0.866 | 0.879 | 0.892 | 0.892 | 0.877 |
|  | RUN7 | 0.987 | 0.987 | 0.986 | 0.981 | 0.987 | 0.885 | 0.878 | 0.883 | 0.865 | 0.876 |
|  | RUN8 | 0.987 | 0.984 | 0.987 | 0.985 | 0.985 | 0.879 | 0.867 | 0.88 | 0.878 | 0.874 |
|  | RUN9 | 0.987 | 0.984 | 0.986 | 0.988 | 0.987 | 0.876 | 0.865 | 0.874 | 0.89 | 0.875 |
|  | RUN10 | 0.987 | 0.984 | 0.986 | 0.986 | 0.984 | 0.876 | 0.871 | 0.864 | 0.878 | 0.879 |

**Table S2.** *Opuntia ficus-indica* individual model TSS and ROC scores across all model repeats, SDM scenarios and algorithms.

| ***Euphorbia tirucalli*** | | **ROC** | | | | | **TSS** | | | | |
| --- | --- | --- | --- | --- | --- | --- | --- | --- | --- | --- | --- |
| **SDM 1** | | **PA1** | **PA2** | **PA3** | **PA4** | **PA5** | **PA1** | **PA2** | **PA3** | **PA4** | **PA5** |
| **RF** | RUN1 | 0.996 | 0.994 | 0.997 | 0.994 | 0.996 | 0.962 | 0.923 | 0.959 | 0.917 | 0.941 |
|  | RUN2 | 0.993 | 0.996 | 0.997 | 0.997 | 0.992 | 0.947 | 0.932 | 0.95 | 0.953 | 0.941 |
|  | RUN3 | 0.992 | 0.996 | 0.997 | 0.996 | 0.992 | 0.941 | 0.935 | 0.953 | 0.929 | 0.926 |
|  | RUN4 | 0.999 | 0.99 | 0.994 | 0.996 | 0.992 | 0.956 | 0.92 | 0.938 | 0.938 | 0.923 |
|  | RUN5 | 0.998 | 0.993 | 0.996 | 0.993 | 0.998 | 0.962 | 0.926 | 0.959 | 0.941 | 0.95 |
|  | RUN6 | 0.996 | 0.993 | 0.999 | 0.996 | 0.996 | 0.956 | 0.938 | 0.967 | 0.953 | 0.932 |
|  | RUN7 | 0.996 | 0.993 | 0.998 | 0.994 | 0.995 | 0.95 | 0.923 | 0.962 | 0.935 | 0.926 |
|  | RUN8 | 0.996 | 0.993 | 0.993 | 0.995 | 0.994 | 0.938 | 0.932 | 0.938 | 0.926 | 0.929 |
|  | RUN9 | 0.996 | 0.993 | 0.995 | 0.996 | 0.995 | 0.95 | 0.923 | 0.935 | 0.941 | 0.932 |
|  | RUN10 | 0.998 | 0.996 | 0.993 | 0.995 | 0.996 | 0.959 | 0.944 | 0.917 | 0.944 | 0.956 |
| **GBM** | RUN1 | 0.994 | 0.993 | 0.995 | 0.993 | 0.995 | 0.95 | 0.908 | 0.95 | 0.905 | 0.935 |
|  | RUN2 | 0.994 | 0.993 | 0.996 | 0.997 | 0.993 | 0.941 | 0.914 | 0.944 | 0.947 | 0.932 |
|  | RUN3 | 0.993 | 0.995 | 0.996 | 0.995 | 0.99 | 0.938 | 0.929 | 0.938 | 0.929 | 0.917 |
|  | RUN4 | 0.998 | 0.989 | 0.994 | 0.995 | 0.991 | 0.947 | 0.911 | 0.926 | 0.938 | 0.911 |
|  | RUN5 | 0.998 | 0.993 | 0.996 | 0.993 | 0.997 | 0.959 | 0.929 | 0.953 | 0.935 | 0.941 |
|  | RUN6 | 0.996 | 0.993 | 0.998 | 0.996 | 0.994 | 0.956 | 0.923 | 0.956 | 0.944 | 0.923 |
|  | RUN7 | 0.994 | 0.992 | 0.998 | 0.992 | 0.994 | 0.941 | 0.908 | 0.953 | 0.917 | 0.92 |
|  | RUN8 | 0.994 | 0.992 | 0.993 | 0.995 | 0.992 | 0.932 | 0.917 | 0.932 | 0.923 | 0.92 |
|  | RUN9 | 0.996 | 0.992 | 0.995 | 0.996 | 0.993 | 0.944 | 0.917 | 0.926 | 0.935 | 0.914 |
|  | RUN10 | 0.996 | 0.996 | 0.989 | 0.995 | 0.995 | 0.962 | 0.938 | 0.896 | 0.935 | 0.938 |
| **SDM 2** | | **ROC** | | | | | **TSS** | | | | |
| **RF** | RUN1 | 0.988 | 0.989 | 0.992 | 0.992 | 0.995 | 0.902 | 0.917 | 0.905 | 0.92 | 0.947 |
|  | RUN2 | 0.992 | 0.99 | 0.993 | 0.99 | 0.994 | 0.917 | 0.911 | 0.923 | 0.911 | 0.92 |
|  | RUN3 | 0.993 | 0.991 | 0.989 | 0.993 | 0.985 | 0.92 | 0.917 | 0.917 | 0.929 | 0.899 |
|  | RUN4 | 0.993 | 0.995 | 0.992 | 0.992 | 0.992 | 0.917 | 0.941 | 0.92 | 0.929 | 0.92 |
|  | RUN5 | 0.994 | 0.993 | 0.991 | 0.991 | 0.992 | 0.926 | 0.932 | 0.911 | 0.923 | 0.92 |
|  | RUN6 | 0.994 | 0.993 | 0.995 | 0.993 | 0.989 | 0.926 | 0.923 | 0.926 | 0.917 | 0.891 |
|  | RUN7 | 0.993 | 0.99 | 0.989 | 0.99 | 0.989 | 0.92 | 0.905 | 0.902 | 0.914 | 0.911 |
|  | RUN8 | 0.994 | 0.99 | 0.991 | 0.996 | 0.992 | 0.92 | 0.905 | 0.905 | 0.935 | 0.899 |
|  | RUN9 | 0.991 | 0.992 | 0.993 | 0.993 | 0.991 | 0.908 | 0.929 | 0.926 | 0.935 | 0.917 |
|  | RUN10 | 0.994 | 0.994 | 0.989 | 0.991 | 0.991 | 0.929 | 0.935 | 0.899 | 0.932 | 0.911 |
| **GBM** | RUN1 | 0.984 | 0.985 | 0.988 | 0.991 | 0.994 | 0.885 | 0.87 | 0.882 | 0.896 | 0.935 |
|  | RUN2 | 0.988 | 0.989 | 0.991 | 0.988 | 0.992 | 0.885 | 0.896 | 0.905 | 0.893 | 0.926 |
|  | RUN3 | 0.99 | 0.987 | 0.987 | 0.991 | 0.985 | 0.905 | 0.893 | 0.908 | 0.905 | 0.896 |
|  | RUN4 | 0.987 | 0.993 | 0.989 | 0.99 | 0.989 | 0.893 | 0.896 | 0.914 | 0.926 | 0.902 |
|  | RUN5 | 0.991 | 0.988 | 0.988 | 0.991 | 0.989 | 0.896 | 0.896 | 0.893 | 0.917 | 0.905 |
|  | RUN6 | 0.991 | 0.99 | 0.991 | 0.99 | 0.986 | 0.917 | 0.908 | 0.905 | 0.905 | 0.891 |
|  | RUN7 | 0.991 | 0.988 | 0.987 | 0.989 | 0.986 | 0.917 | 0.896 | 0.888 | 0.911 | 0.888 |
|  | RUN8 | 0.99 | 0.986 | 0.985 | 0.992 | 0.987 | 0.911 | 0.891 | 0.885 | 0.914 | 0.896 |
|  | RUN9 | 0.989 | 0.989 | 0.989 | 0.991 | 0.99 | 0.899 | 0.911 | 0.908 | 0.917 | 0.914 |
|  | RUN10 | 0.991 | 0.99 | 0.985 | 0.991 | 0.989 | 0.911 | 0.902 | 0.899 | 0.911 | 0.899 |
| **SDM 3** | | **ROC** | | | | | **TSS** | | | | |
| **RF** | RUN1 | 0.992 | 0.992 | 0.991 | 0.995 | 0.99 | 0.945 | 0.942 | 0.926 | 0.933 | 0.915 |
|  | RUN2 | 0.991 | 0.991 | 0.994 | 0.996 | 0.99 | 0.917 | 0.942 | 0.949 | 0.949 | 0.924 |
|  | RUN3 | 0.994 | 0.987 | 0.99 | 0.993 | 0.991 | 0.929 | 0.926 | 0.915 | 0.922 | 0.935 |
|  | RUN4 | 0.988 | 0.994 | 0.993 | 0.995 | 0.988 | 0.924 | 0.938 | 0.938 | 0.938 | 0.926 |
|  | RUN5 | 0.992 | 0.993 | 0.988 | 0.993 | 0.993 | 0.935 | 0.933 | 0.91 | 0.942 | 0.94 |
|  | RUN6 | 0.996 | 0.995 | 0.991 | 0.992 | 0.996 | 0.959 | 0.952 | 0.924 | 0.933 | 0.949 |
|  | RUN7 | 0.995 | 0.987 | 0.995 | 0.991 | 0.987 | 0.938 | 0.922 | 0.956 | 0.933 | 0.917 |
|  | RUN8 | 0.992 | 0.995 | 0.991 | 0.992 | 0.992 | 0.947 | 0.949 | 0.917 | 0.938 | 0.935 |
|  | RUN9 | 0.993 | 0.985 | 0.989 | 0.991 | 0.992 | 0.933 | 0.924 | 0.931 | 0.931 | 0.931 |
|  | RUN10 | 0.994 | 0.992 | 0.989 | 0.988 | 0.993 | 0.949 | 0.926 | 0.915 | 0.917 | 0.924 |
| **GBM** | RUN1 | 0.992 | 0.991 | 0.989 | 0.993 | 0.99 | 0.94 | 0.935 | 0.922 | 0.919 | 0.901 |
|  | RUN2 | 0.991 | 0.992 | 0.994 | 0.995 | 0.99 | 0.912 | 0.945 | 0.942 | 0.947 | 0.919 |
|  | RUN3 | 0.992 | 0.988 | 0.988 | 0.992 | 0.991 | 0.919 | 0.917 | 0.917 | 0.929 | 0.915 |
|  | RUN4 | 0.985 | 0.994 | 0.993 | 0.992 | 0.991 | 0.908 | 0.947 | 0.94 | 0.926 | 0.926 |
|  | RUN5 | 0.99 | 0.992 | 0.986 | 0.994 | 0.993 | 0.924 | 0.929 | 0.912 | 0.945 | 0.94 |
|  | RUN6 | 0.995 | 0.992 | 0.99 | 0.993 | 0.995 | 0.942 | 0.933 | 0.919 | 0.926 | 0.938 |
|  | RUN7 | 0.993 | 0.986 | 0.994 | 0.991 | 0.987 | 0.933 | 0.929 | 0.935 | 0.931 | 0.915 |
|  | RUN8 | 0.992 | 0.994 | 0.99 | 0.991 | 0.992 | 0.949 | 0.947 | 0.919 | 0.926 | 0.919 |
|  | RUN9 | 0.991 | 0.984 | 0.989 | 0.991 | 0.992 | 0.929 | 0.917 | 0.922 | 0.922 | 0.929 |
|  | RUN10 | 0.994 | 0.993 | 0.988 | 0.987 | 0.99 | 0.947 | 0.929 | 0.901 | 0.912 | 0.926 |
| **SDM 4** | | **ROC** | | | | | **TSS** | | | | |
| **RF** | RUN1 | 0.995 | 0.995 | 0.996 | 0.992 | 0.995 | 0.947 | 0.94 | 0.968 | 0.94 | 0.94 |
|  | RUN2 | 0.996 | 0.994 | 0.994 | 0.996 | 0.994 | 0.949 | 0.94 | 0.949 | 0.954 | 0.949 |
|  | RUN3 | 0.993 | 0.994 | 0.997 | 0.996 | 0.995 | 0.931 | 0.94 | 0.952 | 0.949 | 0.943 |
|  | RUN4 | 0.995 | 0.996 | 0.994 | 0.992 | 0.994 | 0.945 | 0.938 | 0.943 | 0.94 | 0.943 |
|  | RUN5 | 0.994 | 0.993 | 0.995 | 0.991 | 0.995 | 0.952 | 0.933 | 0.949 | 0.933 | 0.945 |
|  | RUN6 | 0.997 | 0.994 | 0.994 | 0.993 | 0.997 | 0.949 | 0.936 | 0.938 | 0.952 | 0.952 |
|  | RUN7 | 0.997 | 0.996 | 0.996 | 0.996 | 0.996 | 0.961 | 0.952 | 0.949 | 0.949 | 0.943 |
|  | RUN8 | 0.995 | 0.988 | 0.994 | 0.995 | 0.996 | 0.94 | 0.929 | 0.947 | 0.936 | 0.949 |
|  | RUN9 | 0.995 | 0.996 | 0.992 | 0.996 | 0.994 | 0.949 | 0.956 | 0.922 | 0.959 | 0.947 |
|  | RUN10 | 0.997 | 0.994 | 0.997 | 0.993 | 0.996 | 0.954 | 0.94 | 0.952 | 0.954 | 0.947 |
| **GBM** | RUN1 | 0.995 | 0.995 | 0.993 | 0.992 | 0.994 | 0.945 | 0.943 | 0.956 | 0.926 | 0.933 |
|  | RUN2 | 0.995 | 0.992 | 0.992 | 0.995 | 0.994 | 0.945 | 0.936 | 0.938 | 0.943 | 0.938 |
|  | RUN3 | 0.991 | 0.993 | 0.995 | 0.993 | 0.994 | 0.929 | 0.938 | 0.933 | 0.926 | 0.943 |
|  | RUN4 | 0.994 | 0.995 | 0.994 | 0.992 | 0.993 | 0.936 | 0.931 | 0.931 | 0.931 | 0.929 |
|  | RUN5 | 0.994 | 0.992 | 0.993 | 0.989 | 0.994 | 0.929 | 0.917 | 0.949 | 0.926 | 0.938 |
|  | RUN6 | 0.996 | 0.992 | 0.993 | 0.992 | 0.996 | 0.94 | 0.933 | 0.933 | 0.936 | 0.947 |
|  | RUN7 | 0.997 | 0.996 | 0.996 | 0.995 | 0.993 | 0.956 | 0.952 | 0.943 | 0.938 | 0.929 |
|  | RUN8 | 0.993 | 0.988 | 0.994 | 0.993 | 0.996 | 0.931 | 0.92 | 0.938 | 0.931 | 0.94 |
|  | RUN9 | 0.995 | 0.994 | 0.989 | 0.995 | 0.992 | 0.943 | 0.945 | 0.91 | 0.945 | 0.933 |
|  | RUN10 | 0.997 | 0.993 | 0.995 | 0.991 | 0.996 | 0.952 | 0.929 | 0.936 | 0.926 | 0.943 |
| **SDM 5** | | **ROC** | | | | | **TSS** | | | | |
| **RF** | RUN1 | 0.989 | 0.993 | 0.992 | 0.991 | 0.991 | 0.915 | 0.94 | 0.929 | 0.929 | 0.926 |
|  | RUN2 | 0.992 | 0.994 | 0.992 | 0.994 | 0.99 | 0.942 | 0.931 | 0.94 | 0.938 | 0.926 |
|  | RUN3 | 0.993 | 0.992 | 0.991 | 0.993 | 0.993 | 0.931 | 0.931 | 0.935 | 0.924 | 0.947 |
|  | RUN4 | 0.989 | 0.99 | 0.993 | 0.994 | 0.993 | 0.935 | 0.915 | 0.924 | 0.931 | 0.945 |
|  | RUN5 | 0.992 | 0.988 | 0.989 | 0.996 | 0.994 | 0.938 | 0.919 | 0.91 | 0.938 | 0.947 |
|  | RUN6 | 0.993 | 0.994 | 0.993 | 0.995 | 0.993 | 0.929 | 0.922 | 0.94 | 0.945 | 0.935 |
|  | RUN7 | 0.993 | 0.993 | 0.994 | 0.993 | 0.993 | 0.933 | 0.931 | 0.947 | 0.935 | 0.956 |
|  | RUN8 | 0.991 | 0.993 | 0.993 | 0.997 | 0.989 | 0.933 | 0.935 | 0.919 | 0.947 | 0.931 |
|  | RUN9 | 0.991 | 0.991 | 0.993 | 0.995 | 0.993 | 0.935 | 0.929 | 0.926 | 0.938 | 0.949 |
|  | RUN10 | 0.992 | 0.99 | 0.994 | 0.994 | 0.986 | 0.931 | 0.926 | 0.942 | 0.926 | 0.906 |
| **GBM** | RUN1 | 0.986 | 0.994 | 0.991 | 0.989 | 0.989 | 0.917 | 0.938 | 0.922 | 0.915 | 0.924 |
|  | RUN2 | 0.991 | 0.992 | 0.991 | 0.994 | 0.99 | 0.931 | 0.915 | 0.926 | 0.94 | 0.926 |
|  | RUN3 | 0.991 | 0.992 | 0.99 | 0.994 | 0.993 | 0.912 | 0.924 | 0.915 | 0.933 | 0.935 |
|  | RUN4 | 0.991 | 0.988 | 0.993 | 0.994 | 0.993 | 0.929 | 0.91 | 0.91 | 0.926 | 0.933 |
|  | RUN5 | 0.991 | 0.985 | 0.988 | 0.994 | 0.994 | 0.926 | 0.912 | 0.908 | 0.926 | 0.947 |
|  | RUN6 | 0.993 | 0.993 | 0.992 | 0.994 | 0.993 | 0.929 | 0.922 | 0.938 | 0.933 | 0.938 |
|  | RUN7 | 0.99 | 0.991 | 0.993 | 0.991 | 0.992 | 0.919 | 0.919 | 0.931 | 0.929 | 0.942 |
|  | RUN8 | 0.99 | 0.99 | 0.991 | 0.997 | 0.987 | 0.924 | 0.931 | 0.91 | 0.94 | 0.915 |
|  | RUN9 | 0.989 | 0.988 | 0.992 | 0.994 | 0.994 | 0.924 | 0.933 | 0.922 | 0.931 | 0.945 |
|  | RUN10 | 0.991 | 0.99 | 0.995 | 0.993 | 0.987 | 0.929 | 0.912 | 0.942 | 0.917 | 0.908 |

**Table S3.** *Euphorbia tirucalli* individual model TSS and ROC scores across all model repeats, SDM scenarios and algorithms.

**Appendix D: Summary statistics for *Opuntia ficus-indica* and *Euphorbia tirucalli* based on individual model TSS and ROC scores**

|  | | ***Opuntia ficus-indica*** | | | ***Euphorbia tirucalli*** | | |
| --- | --- | --- | --- | --- | --- | --- | --- |
|  |  | **All models** | **By algorithm** | | **All models** | **By algorithm** | |
|  |  |  | **RF** | **GBM** |  | **RF** | **GBM** |
| **TSS** | **Minimum** | 0.85 | 0.884 | 0.85 | 0.87 | 0.891 | 0.87 |
|  | **Maximum** | 0.942 | 0.942 | 0.927 | 0.968 | 0.968 | 0.962 |
|  | **Mean** | 0.898 | 0.910 | 0.886 | 0.929 | 0.934 | 0.925 |
| **ROC** | **Minimum** | 0.981 | 0.988 | 0.981 | 0.984 | 0.985 | 0.984 |
|  | **Maximum** | 0.996 | 0.996 | 0.995 | 0.999 | 0.999 | 0.998 |
|  | **Mean** | 0.990 | 0.992 | 0.988 | 0.992 | 0.993 | 0.992 |

**Table S4.** Mean, minimum and maximum TSS and ROC scores across *Opuntia ficus-indica* and *Euphorbia tirucalli* SDMs.

| **SDM Scenario** | **Ensemble weighted mean by TSS** | | | | | | | |
| --- | --- | --- | --- | --- | --- | --- | --- | --- |
|  | ***Opuntia ficus-indica*** | | | | ***Euphorbia tirucalli*** | | | |
|  | TSS | Cutoff | Sensitivity | Specificity | TSS | Cut-off | Sensitivity | Specificity |
| **1** | **0.930** | **53.9** | **96.689** | **96.338** | **0.955** | **62.9** | **97.754** | **97.706** |
| 2 | 0.914 | 51.9 | 96.097 | 95.264 | 0.932 | 54.1 | 97.393 | 95.767 |
| 3 | 0.916 | 49.1 | 96.54 | 95.006 | 0.948 | 50.4 | 98.341 | 96.476 |
| 4 | 0.925 | 57.7 | 96.063 | 96.397 | 0.954 | 64.8 | 97.792 | 97.662 |
| 5 | 0.918 | 55.7 | 95.683 | 96.144 | 0.949 | 46.6 | 98.527 | 96.371 |
| **SDM Scenario** | **Ensemble weighted mean by ROC** | | | | | | | |
|  | ***Opuntia ficus-indica*** | | | | ***Euphorbia tirucalli*** | | | |
|  | ROC | Cutoff | Sensitivity | Specificity | ROC | Cut-off | Sensitivity | Specificity |
| **1** | **0.997** | **54.1** | **96.689** | **96.382** | **0.998** | **63.1** | **97.754** | **97.730** |
| 2 | 0.994 | 52.6 | 96.06 | 95.359 | 0.996 | 55.8 | 97.275 | 96.027 |
| 3 | 0.995 | 49.3 | 96.54 | 95.095 | 0.997 | 51.2 | 98.341 | 96.531 |
| **4** | **0.996** | **55.8** | **96.063** | **96.441** | **0.998** | **64.8** | **97.792** | **97.662** |
| 5 | 0.995 | 55.9 | 95.683 | 96.181 | 0.997 | 47.0 | 98.527 | 96.408 |

**Table S5.** Ensemble model evaluation metrics and binary cut-off points per SDM scenario and by specie.

**
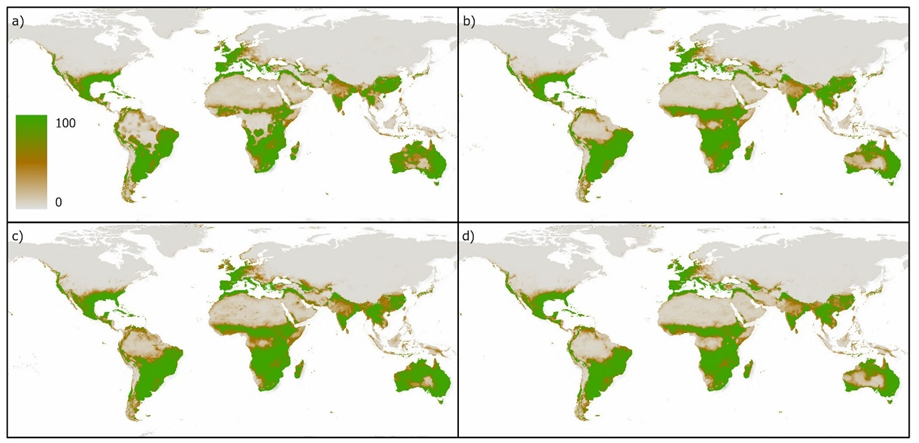
Appendix E: SDM scenarios 2-5 ensemble projections**

**Figure S5.** Ensemble output projections for SDM scenarios 2-5 (a-d) based on current observations of *O. ficus-indica*.


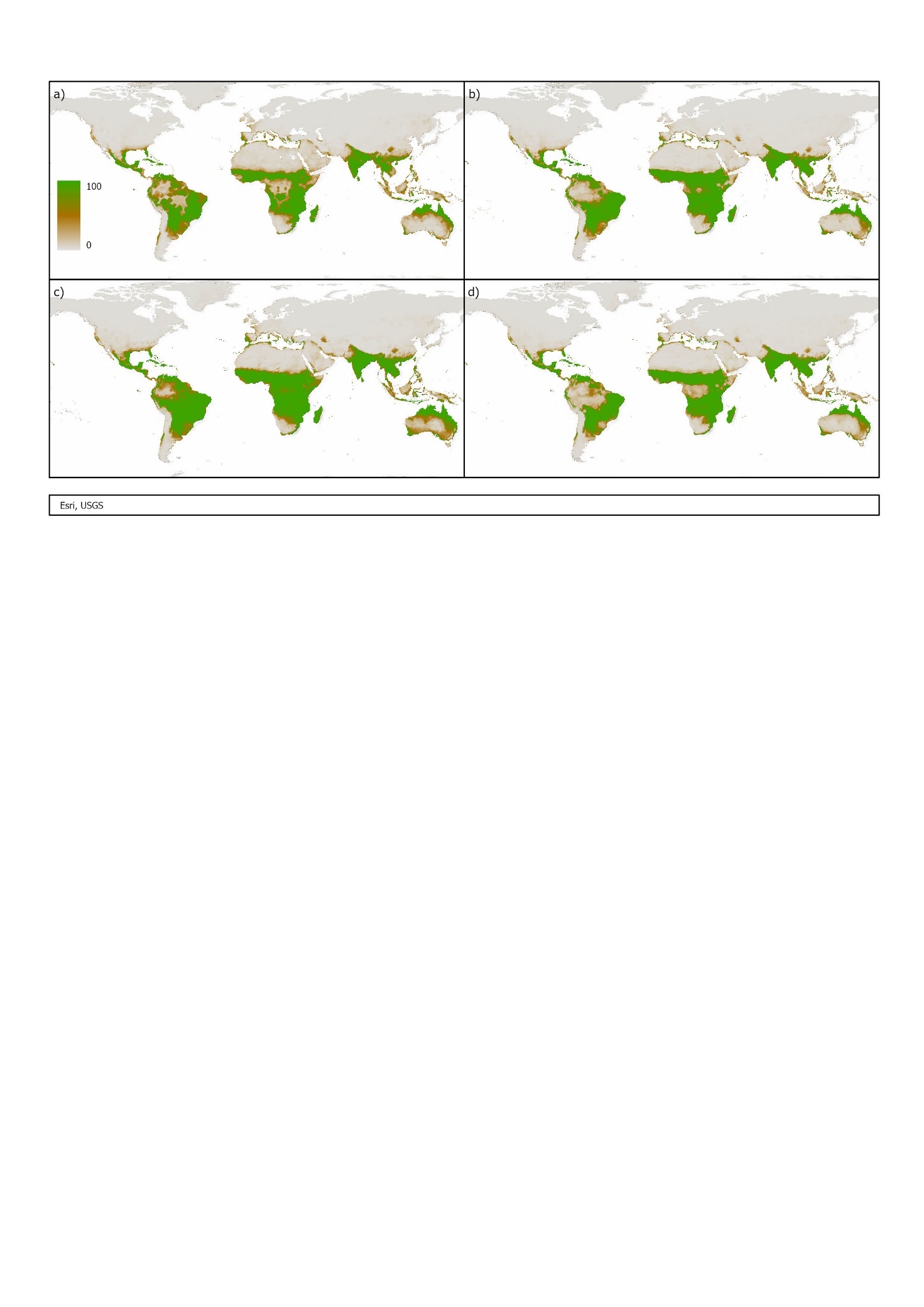


**Figure S6.** Ensemble output projections for SDM scenarios 2-5 (a-d) based on current observations of *E. tirucalli*.

**Appendix F: SDM scenarios 1-5 TSS vs ROC scatter plots**


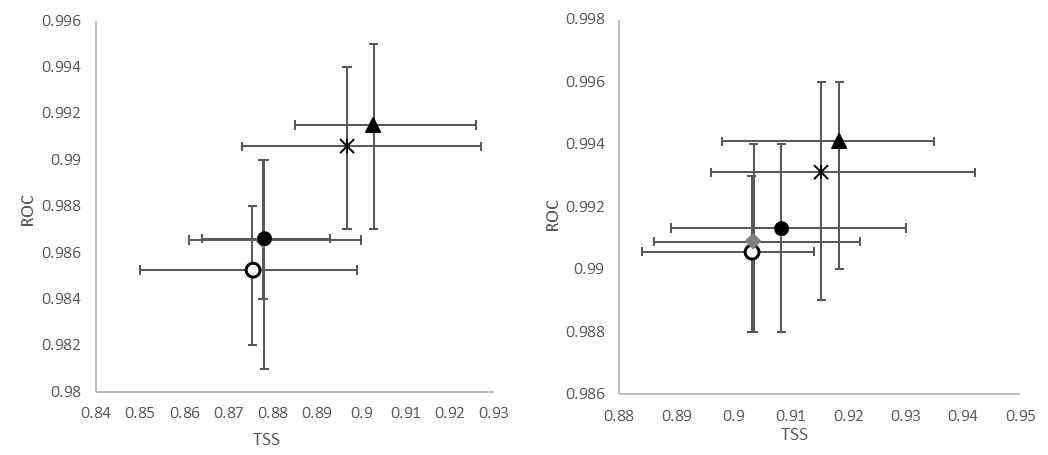


**Figure S7.** TSS vs ROC scatter plots for all individual models of *O. ficus-indica* (GBM left, RF right) by SDM scenario: SDM 1 – triangle, SDM 2 – open circle, SDM 3 – grey diamond, SDM 4 - star , SDM 5 – circle.

***
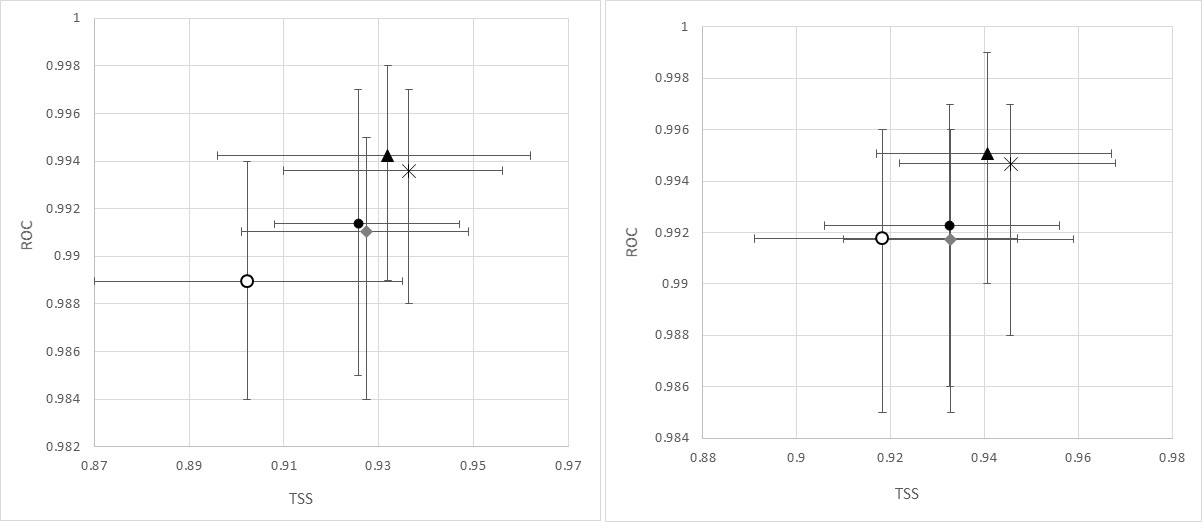
***

**Figure S8.** TSS vs ROC scatter plots for all individual models of *E. tirucalli* (GBM left, RF right) by SDM scenario: SDM 1 – triangle, SDM 2 – open circle, SDM 3 – grey diamond, SDM 4 - star , SDM 5 – circle.
